# ECM characterization and 3D bioprinted models of NSCLC for investigating stiffness-dependent tumor behavior and drug response

**DOI:** 10.1016/j.mtbio.2025.101823

**Published:** 2025-04-30

**Authors:** Shiwei Xu, Xin Sun, Yexin Gu, Tong Liu, Shiyin Liu, Yuan Weng, Weimin Zhang, Leisheng Wang, Mengzhen Zhou, Guye Lu, Min Tang, Haifeng Wang, Jinyou Li

**Affiliations:** a Department of Thoracic Surgery, Afflicated Hospital of Jiangnan University, Wuxi, 214122, China; b Wuxi School of Medicine, Jiangnan University, Wuxi, 214000, China; c Jiangsu Hansoh Pharmaceutical, Shanghai, 200120, China; d Cyberiad Biotechnology, Shanghai, 201112, China; e School of Medicine, Southeast University, Nanjing, 210009, China; f Institute of Interdisciplinary Integrative Medicine Research, Shanghai University of Traditional Chinese Medicine, Shanghai, 201203, China; g Department of Thoracic Surgery, Shanghai Pulmonary Hospital, Shanghai, 200433, China; h Department of Pulmonary Medicine, Wuxi People's Hospital, Wuxi, 214023, China

**Keywords:** 3D bioprinting, Non-small cell lung cancer, Extracellular matrix, Stiffness, Patient-derived tissue

## Abstract

The heterogeneity and complex extracellular matrix (ECM) characteristics of non-small cell lung cancer (NSCLC) present significant challenges for understanding its pathological mechanisms and advancing precise treatment strategies. This study characterized the physicochemical properties of native NSCLC ECM to inform the biomimetic design of 3D models utilizing biomaterials and light-based 3D bioprinting technologies. A tunable 3D model was constructed that replicates the interfacial structures and matrix stiffness of both lung cancer tissue and adjacent normal tissue. This model elucidates the impact of matrix stiffness on cellular behaviors, including proliferation, invasion, and drug sensitivity, and delineates the role of the CCN1 gene under different mechanical conditions. Specifically, it demonstrates that a reduction in CCN1 expression within soft matrices can attenuate the migratory and proliferative capabilities of tumor cells. Furthermore, primary NSCLC patient-derived bioprinted tissues validated the model fidelity to clinical samples and its predictive potential for responses to combined chemotherapy and immunotherapy. This study establishes a versatile platform for NSCLC modeling and research, advancing biomaterial and bioprinting strategies to replicate the tumor microenvironment and optimize therapeutic approaches.

## Graphical abstract

**Figure:**
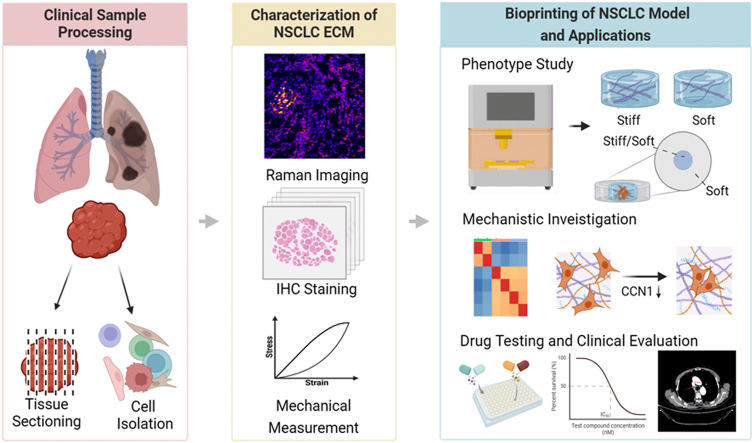

## 1 Introduction

Lung cancer (LC) remains the leading cause of cancer-related mortality in men and the second leading cause in women worldwide [1]. Lung cancer is classified into small cell lung cancer (SCLC) and non-small cell lung cancer (NSCLC). NSCLC accounts for over 80 % of all LC cases [2]. NSCLC is further divided, based on histological differences, into adenocarcinoma, squamous cell carcinoma, large cell lung cancer, neuroendocrine tumors, among others, with adenocarcinoma representing over 50 % [3]. The differentiation level of tumor cells is often linked to treatment outcomes. Clinically, lower differentiation is associated with greater malignancy and worse treatment results, whereas higher differentiation indicates better therapeutic effects [4]. Despite advances in diagnosis and treatment, only about 15 % of NSCLC patients present with early-stage disease suitable for surgical resection, while more than half are diagnosed at advanced stages [5]. Metastasis is the primary cause of mortality in these patients [6]. For advanced NSCLC, treatment options primarily include radiotherapy, chemotherapy, targeted therapy, and immunotherapy [7]. While chemotherapy, targeted therapy, and immune checkpoint inhibitors have shown efficacy in some patients, approximately 30 % fail to respond, highlighting the critical need for personalized therapeutic strategies [8,9].

Treatment decisions in lung cancer often rely on clinical and molecular diagnostics, which fail to capture the intricate complexities of the tumor microenvironment (TME) [10,11]. Traditional 2D cell culture systems are insufficient for replicating the architecture and physiochemical properties of the TME, including the extracellular matrix (ECM) and its critical roles in cell behavior and drug penetration [12,13]. To address these limitations, 3D in vitro tissue models have emerged as valuable tools for studying tumor biology and enhancing the accuracy of drug screening [[14], [15], [16]]. These models utilize cells and biomaterials to mimic the compositional, morphological, structural, and functional characteristics of native tissues, as well as the specific microenvironmental conditions found in vivo [17,18].

Several approaches have been developed for constructing 3D models, including stem cell-derived organoid technology, organ-on-a-chip systems employing microfluidics, and 3D bioprinting [[19], [20], [21], [22]]. These methodologies have been applied to various cancers, such as breast, colorectal, liver, and brain tumors. Among these, tumor organoids cultured in Matrigel are the most extensively studied [[23], [24], [25]]. However, Matrigel has notable limitations, including inadequate stiffness for modeling tumor tissues, inability to form specific architecture, and batch-to-batch variability, which hinder its suitability for replicating the TME with precise ECM characteristics [[26], [27], [28]]. In contrast, light-based 3D bioprinting technology combined with photosensitive biomaterials offers a highly controllable platform for simulating the native architecture and ECM [[29], [30], [31]]. This approach enables precise manipulation of ECM composition, stiffness, and interfacial structures, facilitating the creation of models tailored to specific TME characteristics [32]. Additionally, 3D bioprinting supports the co-culture of multiple cell types, providing a more comprehensive representation of patient-specific tumor tissues [33]. While such advancements have shown great promise in developing patient-derived in vitro drug screening platforms for various cancers including NSCLC and others [25,[34], [35], [36]], their potential application in investigating mechanical regulation in NSCLC remains largely unexplored.

Here, we developed a 3D bioprinted model of NSCLC using digital light processing (DLP)-based 3D bioprinting. The bio-ink formulation included methacrylated gelatin (GelMA), methacrylated hyaluronic acid (HAMA), and the photoinitiator lithium phenyl-2,4,6-trimethylbenzoylphosphinate (LAP) to mimic the ECM components and stiffness of NSCLC tissue and the adjacent normal tissue. Transcriptomic analysis revealed significant differences in gene expression profiles between tumor cells cultured in 2D versus 3D systems. Further investigation revealed that softer ECM environments enhanced tumor cell invasiveness and proliferation, whereas stiffer ECM models were associated with increased resistance to chemotherapy but not targeted therapies. Building on these findings, we developed patient-derived tissue (PDT) models that preserved the cellular heterogeneity of clinical samples, encompassing tumor cells, CD45^+^ immune cells, CD3^+^ T cells, and other cell populations. These models also retained the molecular profiles of the original patient tissues, providing a more accurate representation of the tumor microenvironment. PDT models were subsequently employed to assess the efficacy of combination therapy with chemotherapeutic agents and immunotherapy, with experimental outcomes demonstrating strong concordance with clinical treatment responses. These results underscore the utility of 3D bioprinting technology as a robust platform for investigating the regulatory effects of biomimetic ECM properties and matrix stiffness on lung cancer progression. Moreover, PDT models constructed via this approach show substantial promise for guiding personalized therapeutic strategies, offering a practical tool for tailoring treatments to individual patient profiles in clinical settings.

## 2 Result

### 2.1 Characterization of physiochemical property of NSCLC ECM and generation of 3D bioprinted LC model

**Fig. 1:**
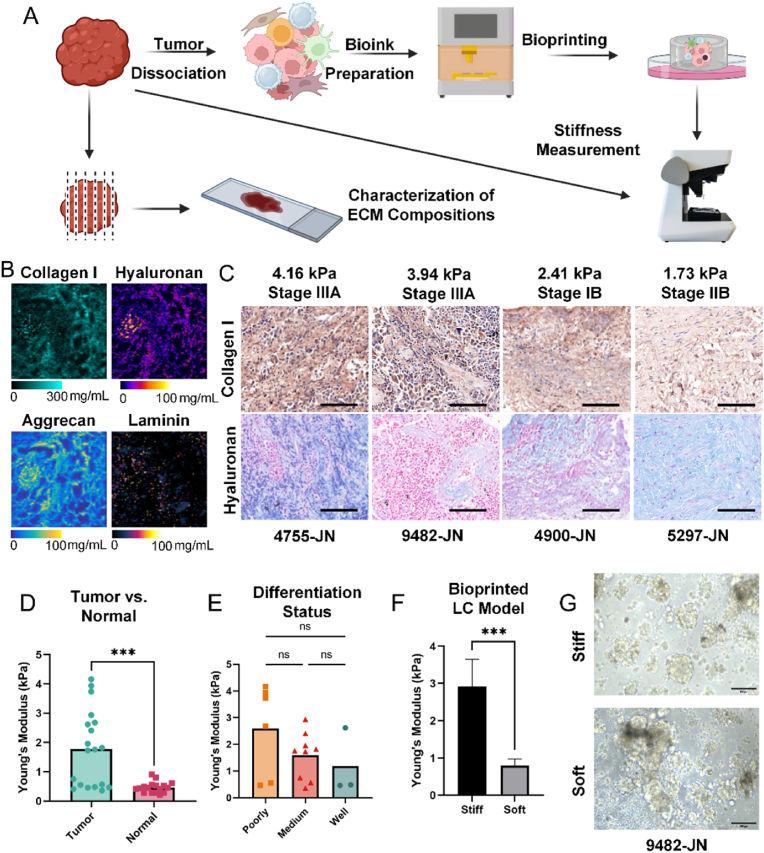
**Characterization of the physicochemical properties of NSCLC ECM and generation of 3D bioprinted lung cancer (LC) models.** **(A)** Schematic workflow showing tumor tissue processing, ECM component and stiffness analysis, and 3D bioprinting of LC models. (B) Representative Raman images showing the distribution of ECM components in NSCLC tumor tissue, including collagen I, hyaluronan, aggrecan, and laminin. Image size: 1 mm × 1 mm. **(C)** Immunohistochemical staining for type I collagen and hyaluronan in representative LUAD patient tissue specimens. Scale bar: 100 μm. **(D)** Stiffness measurements of primary tumor and adjacent normal tissues from 16 NSCLC patients. **(E)** Analysis of tumor stiffness across different histological differentiation statuses (poorly, moderately, and well differentiated). **(F)** Stiffness measurements of 3D bioprinted soft and stiff LC models. **(G)** Bright-field images of primary cells derived from LUAD patients cultured in 3D bioprinted models. Scale bar: 100 μm. Data are presented as mean ± SD. Statistical significance: ∗∗∗p < 0.001; ns, not significant.

To guide the design of bioinks and optimize 3D printing parameters, we first characterized the compositional and mechanical properties of the ECM of NSCLC tissues. Clinical samples, comprising 17 pairs of NSCLC tissues and corresponding peritumoral tissues, were analyzed (Fig. 1A, Table 1). Raman imaging and immunohistochemical staining of representative tumor samples across clinical stages revealed prominent presence of type I collagen, hyaluronan, aggrecan, and laminin (Fig. 1B and C, Supplementary Figures 1A). In addition, collagen I expression positively correlated with tumor stiffness but not with clinical staging. A significant difference in stiffness was observed between the tumor tissue and the peritumoral tissue. Tumor tissues exhibited high heterogeneity in stiffness, ranging from 0.4 to 4 kPa, while peritumoral tissues displayed more uniform stiffness, averaging 0.46 kPa (Fig. 1D). This variability highlights the diverse microenvironments within tumors. Samples were further classified by pathological differentiation, including poorly, moderately, and well-differentiated. A trend toward reduced stiffness with increasing differentiation was observed, though differences were not statistically significant due to limited sample size (Fig. 1E). Analysis by TNM stage and histological subtype, including lung adenocarcinoma (LUAD) and non-LUAD subtypes, showed no significant stiffness differences (Supplementary Fig. 1B–C). Additional analyses categorized by clinical and demographic parameters—such as sex, age, recurrence status, and postoperative treatment—demonstrated that patients who received postoperative treatment, typically indicated clinically as high-risk, tended to exhibit higher tumor stiffness compared to lower-risk patients (Supplementary Fig. 1D–G).

**Table 1:** Patient demographics, sample characteristics, and treatment follow-up.

Sample ID	Age	Sex	Tumor (kPa)	Normal (kPa)	Subtype	Differentiation Status	TNM Classification	Drug treatment	Follow-up
9883-JN	73	F	1.82	0.46	Adenocarcinoma	Moderately differentiated	T2aN_0_M_0_ Stage ⅠB	Untreated	No recurrence
0445-JN	51	F	1.96	0.46	Adenocarcinoma	Moderately differentiated	T1_c_N_0_M_0_ Stage ⅠA_3_	Untreated	No recurrence
0730-JN	59	M	1.76	0.52	Adenocarcinoma	Moderately differentiated	T1_c_N_0_M_0_ Stage ⅠA_3_	Untreated	No recurrence
4487-JN	71	F	2.94	0.29	Adenocarcinoma	Moderately differentiated	T2_a_N_0_M_0_ Stage ⅠB	Untreated	No recurrence
4755-JN	55	M	4.16	0.42	Adenocarcinoma	poorly differentiated	T2_a_N_2_M_0_ Stage ⅢA	Chemotherapy and immunotherapy	Recurrence
9625-JN	60	F	0.48	0.44	Adenocarcinoma	Well differentiated	T1_b_N_0_M_0_ Stage ⅠA_2_	Untreated	No recurrence
4900-JN	66	F	2.41	0.80	Adenocarcinoma	Moderately differentiated	T2_a_N_0_M_0_ Stage ⅠB	Chemotherapy	No recurrence
6167-JN	65	M	2.62	0.91	Adenocarcinoma	Well differentiated	T1_b_N_0_M_0_ Stage ⅠA_2_	Targeted therapy	No recurrence
4275-JN	57	F	0.75	0.61	Squamous cell carcinoma	Moderately differentiated	T1_c_N_0_M_0_ Stage ⅠA_3_	Untreated	No recurrence
0240-JN	66	M	0.55	0.56	Adenocarcinoma	poorly differentiated	T_4_N_2_M_0_ Stage ⅢB	Chemotherapy and immunotherapy	Recurrence
4513-JN	76	M	0.41	0.28	Giant cell carcinoma	–	T_3_N_0_M_0_ Stage ⅡB	Chemotherapy and immunotherapy	Recurrence
6512-JN	72	F	–	–	Adenocarcinoma	poorly differentiated	T_3_N_0_M_0_ Stage ⅡB	Untreated	Recurrence
7029-JN	75	M	–	–	Adenocarcinoma	Moderately differentiated	T_2a_N_0_M_0_ Stage ⅠB	Untreated	No recurrence
5047-JN	74	F	0.57	0.28	Adenocarcinoma	Moderately differentiated	T_1b_N_0_M_0_ Stage ⅠA_2_	Untreated	No recurrence
4466-JN	77	M	0.47	0.49	Adenocarcinoma	poorly differentiated	T_2a_N_2_M_0_ Stage ⅢA	Untreated	No recurrence
4139-JN	71	M	0.46	0.42	Adenocarcinoma	Moderately to well differentiated	T1_c_N_0_M_0_ Stage ⅠA_3_	Untreated	No recurrence
5297-JN	62	M	1.73	0.29	Adenocarcinoma	Moderately differentiated	T3N_0_M_0_ Stage ⅡB	Untreated	No recurrence
9139-JN	39	F	0.36	0.53	Adenocarcinoma	Moderately differentiated	T1_c_N_0_M_0_ Stage ⅠA_3_	Untreated	No recurrence
9482-JN	83	M	3.94	0.21	Adenocarcinoma	Poorly to moderately differentiated	T_2a_N_2_M_0_ Stage ⅢA	Immunotherapy	Recurrence
9012-JN	71	M	2.68	–	Adenocarcinoma	poorly differentiated	T_2a_N_0_M_0_ Stage ⅠB	Chemotherapy	No recurrence
7114-JN	71	F	3.74	–	Adenocarcinoma	Poorly to moderately differentiated	T_2a_N_2_M_0_ Stage ⅢA	Immunotherapy	No recurrence

The ECM characterization informed the design of bioinks mimicking NSCLC ECM. Given the established abundance and significant biological roles of type I collagen and hyaluronan in NSCLC progression, these two ECM constituents were selected as the primary bioink materials for subsequent experiments in this study [37]. GelMA was selected to replicate the collagen component, HAMA mimicked the polysaccharide content. Successful methacrylation ensured the natural materials to be photopolymerizable (Supplementary Fig. 1H–I). By modulating biomaterial concentrations and adjusting parameters such as light intensity and exposure time during crosslinking, hydrogel stiffness was tailored to match poorly and well-differentiated tumor tissues. Specifically, the photoinitiator LAP concentration and exposure conditions were optimized to control crosslinking density (Table 2). Two stiffness models were developed: one mimicking the average stiffness of well-differentiated tumor tissues (soft model) and the other reflecting the average stiffness of poorly differentiated tumor tissues (stiff model) (Fig. 1F).

**Table 2:** Bioink formulation and printing parameters.

Group	Bioink Formulation	Photoinitiator Concentration	Printing parameter: Light Intensity	Printing Parameter: Exposure Time
Soft	GELMA 3.86 %, HAMA 0.25 %	0.1 %	150 mW/cm^2^	20 s
Stiff		0.2 %	80 mW/cm^2^	16 s

After bioink formulation and printing optimization, patient-derived tumor cells were homogenously mixed with the bioink. Using a DLP 3D bioprinter, we fabricated 3D NSCLC models. The cells demonstrated robust growth in both the soft and stiff models, confirming their biocompatibility for LC modeling and culture (Fig. 1G).

### 2.2 Bioprinted hydrogel culture enriched cancer-relevant pathways in LC cells

**Fig. 2:**
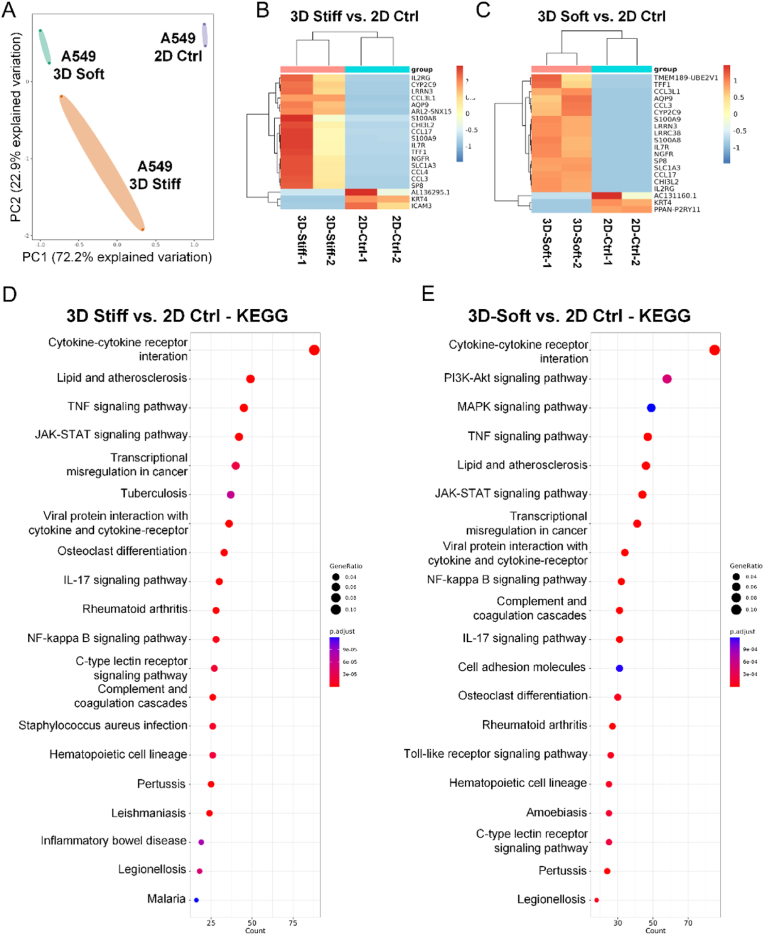
**Bioprinted hydrogel culture enriched cancer relevant pathways in LC cells.** **(A)** Principal component analysis (PCA) plots of the transcriptome of A549 cells cultured under 2D, 3D soft, and 3D stiff conditions (n = 2). **(B)** Heatmap of the top 20 differentially expressed genes in A549 cells cultured in the 3D stiff model compared to the 2D culture group. **(C)** Heatmap of the top 20 differentially expressed genes in A549 cells cultured in the 3D soft model compared to the 2D culture group. **(D)** KEGG pathway enrichment analysis for genes with increased expression in A549 cells cultured in the 3D stiff model compared to the 2D culture group. **(F)** KEGG pathway enrichment analysis for genes with increased expression in A549 cells cultured in the 3D soft model compared to the 2D culture group.

We first investigated how 3D culture conditions alter cell phenotypes compared to traditional 2D culture. Using optimized bioink and 3D bioprinting parameters, we cultured a LUAD cell line, A549 cells, in 3D models with varying stiffness for one week, with conventional 2D culture serving as a control. RNA sequencing was performed to analyze the transcriptomic profiles of A549 cells under these conditions. Principal component analysis (PCA) revealed a distinct transcriptional profile for 2D cultured cells compared to any 3D culture condition (Fig. 2A), emphasizing the transformative impact of 3D culture conditions on the transcriptional landscape of tumor cells.

Comparative analysis revealed the superior ability of 3D cultures to enrich genes associated with tumor progression. Notably, in 3D stiff models, 17 of the top 20 differentially expressed genes were significantly upregulated compared to 2D cultures, including key cancer-related genes (Fig. 2B). These genes are implicated in critical processes such as immune modulation (IL2RG, CCL3L1, CCL17, and CCL4), inflammation (S100A8, S100A9), cellular proliferation (NGFR, SP8, and TFF1), and tumor invasiveness (CHI3L2 and AQP9). Similarly, the 3D soft models also demonstrated significant enrichment, with 17 out of 20 top differentially expressed genes being upregulated. These included genes such as TMEM189, TFF1, CCL3L1, AQP9, CCL3, CYP2C9, S100A9, LRRN3, LRRC38, S100A8, IL7R, NGFR, SP8, SLC1A3, CCL17, CHI3L2, and IL2RG (Fig. 2C). Genes overlapping between stiff and soft conditions, such as AQP9, S100A8, S100A9, IL2RG, CCL3L1, CCL17, NGFR, and CHI3L2, further underscore the consistent role of 3D culture in promoting key tumorigenic pathways. The change in gene expression suggested that 3D culture enhanced the expression of genes critical to tumor progression and cellular activity, which were less prominent in 2D culture.

Gene Ontology (GO) and Gene Set Enrichment Analysis (GSEA) revealed significant upregulation of pathways related to cytokine activity and cellular proliferation in 3D cultured cells, reflecting the enhanced cell-cell and cell-matrix interactions unique to 3D environments (Supplementary Fig. 2A–D). KEGG pathway enrichment analysis revealed significant activation of cytokine–cytokine receptor interaction pathways in 3D cultures, suggesting enhanced cell–cell and cell–matrix signaling. Moreover, tumor-associated signaling pathways, including NF-κB, Toll-like receptor, JAK-STAT, and TNF signaling, were significantly enriched in the 3D model. These pathways are known to contribute to tumor progression, inflammatory regulation, and therapeutic resistance (Fig. 2D and E, Supplementary Fig. 2E–F) [[38], [39], [40], [41]]. Together, these findings strengthen the relevance of the 3D culture system in modeling key oncogenic processes.

### 2.3 Stiffness modulated LC cell phenotypes in bioprinted tumor-peritumor model

**Fig. 3:**
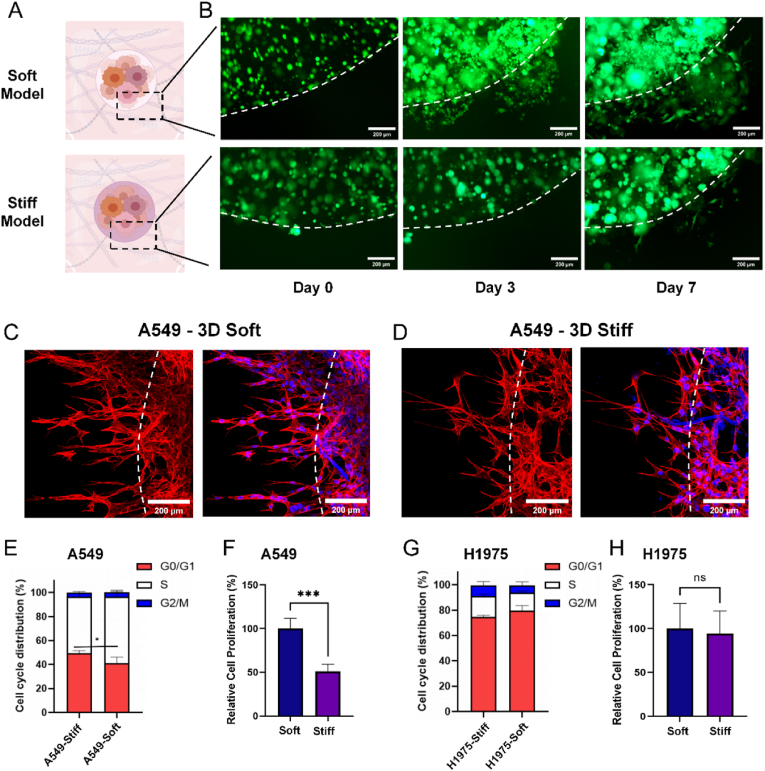
**ECM stiffness modulated LC cell phenotypes in bioprinted tumor-peritumor models.** **(A)** Schematic representation of the 3D tumor-peritumor model illustrating the tumor and normal tissue interface, with varying stiffness in the tumor region. **(B)** Invasion of A549 cells cultured in the two stiffness models at day 0 (immediately after printing), day 3, and day 7. Scale bar: 200 μm. **(C, D)** Confocal microscopy images of the cytoskeleton of invading A549 cells cultured in the two stiffness models, stained with phalloidin. Scale bar: 200 μm. **(E)** Cell cycle and (F) intracellular ATP measurements in A549 cells cultured in the two models. **(G)** Cell cycle and **(H)** intracellular ATP measurements in H1975 cells cultured in the two models. Data are presented as mean ± SD. Statistical significance: ∗p < 0.05, ∗∗∗p < 0.001; ns, not significant.

To investigate how matrix stiffness influences LC cell invasion and proliferation, we next generated bioprinted models using the A549 (EGFR wildtype) and H1975 (EGFR L858R/T790M mutant) cell lines, which were selected to evaluate differential responses to EGFR-targeted therapies in wild-type versus mutant EGFR backgrounds. Bioink containing tumor cells was printed at the center of 24-well plates to simulate tumor tissues of varying stiffness. Acellular regions mimicking soft peritumoral tissue were subsequently bioprinted around the central tumor region to establish a well-defined tumor–peritumor interface, facilitating the observation of tumor cell migration (Fig. 3A)

Invasion dynamics of A549 cells showed a significantly higher rate of outward invasion in the soft model compared to the stiff model (Fig. 3B). Confocal imaging of phalloidin-stained A549 cytoskeletons further supported these findings, revealing markedly greater outward invasion in the soft model (Fig. 3C and D). A similar trend was observed in H1975 cells, which also exhibited enhanced invasion in the soft matrix after two weeks of culture. Imaging at various magnifications consistently demonstrated more pronounced invasion patterns in the softer model for H1975 cells (Supplementary Figure 3). These findings are consistent with previous reports in glioblastoma and cervical cancer, where softer ECM conditions facilitated greater tumor cell motility and invasiveness [42,43]. Our results extend this understanding to lung cancer cells, reinforcing the concept that reduced matrix stiffness promotes cellular movement across diverse tumor types in 3D culture systems.

Next, we analyzed cell cycle distributions and proliferation rates under soft and stiff conditions, conducted with n = 3 replicates. A549 cells displayed a shift in cell cycle dynamics, with significantly fewer cells in the G0/G1 phase and more cells in the G2/M phase in the soft model, suggesting an accelerated cell cycle progression (Fig. 3E). ATP assays showed that A549 cell proliferation rates were approximately 50 % higher in the soft matrix compared to the stiff matrix, consistent with the observed invasion patterns (Fig. 3F). In contrast, H1975 cells did not show significant differences in cell cycle distribution or proliferation rates between the soft and stiff models (Fig. 3G and H). This disparity likely reflects differences in mechanosensitivity between tumor cell subtypes. While the precise mechanisms remain unclear, the EGFR mutation status may contribute to these differences. Specifically, H1975 cells harbor L858R and T790M EGFR mutations, which are known to enhance constitutive EGFR signaling and sustain activation of downstream pathways such as RAS/MAPK/ERK [44]. This sustained signaling could potentially reduce H1975 cells’ dependence on ECM stiffness cues, unlike A549 cells with wild-type EGFR, which appear more responsive to mechanical variations. These findings highlight EGFR status as a potential modulator of tumor cell behavior in mechanical microenvironments, though further studies are needed to clarify this relationship.

We then tested the drug response of cells cultured in the 3D Bioprinted LC Models of both soft & stiff matrices. Furmonertinib, an EGFR-targeted drug, and its bioactive metabolite AST5902 were tested on A549 and H1975 cell line-based 3D LC models, as A549 is an EGFR wildtype lung adenocarcinoma cell while H1975 is an EGFR mutant one. IC_50_ of the drugs to the cells were calculated and compared. The results revealed that H1975 exhibited more sensitive to the targeted drugs than A549 cells as expected, with no apparent differences between soft and stiff models (Supplementary Figure 6A). In contrast, chemotherapy drug responses varied with matrix stiffness. Cells in the soft matrix showed greater sensitivity to cisplatin compared to those in the stiff matrix, suggesting a stiffness-dependent modulation of drug efficacy (Supplementary Fig. 6B). This sensitivity could be related to differential proliferation rates. The low proliferation of cells in the stiff models might have increased their resistance to DNA-targeting agents.

### 2.4 LC cells exhibited distinct transcriptome in 3D stiff and soft models

**Fig. 4:**
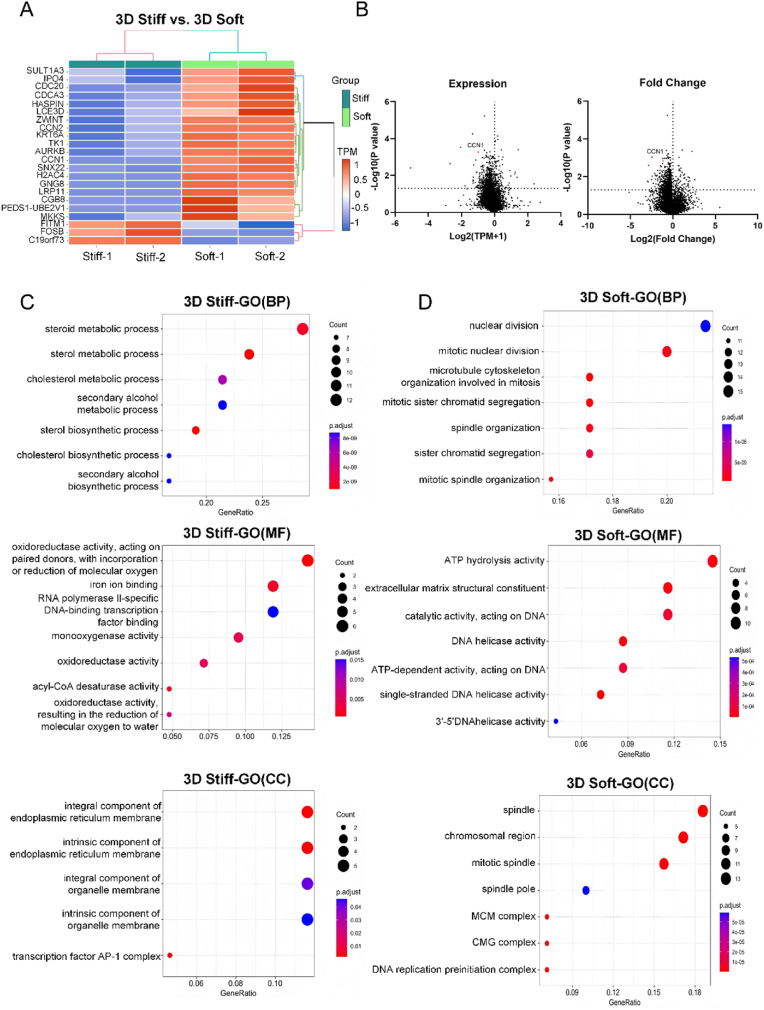
**LC cells exhibited distinct transcriptome in 3D stiff and soft models.** **(A)** Heatmap of top differentially expressed genes (DEGs) between A549 cells cultured in 3D stiff and 3D soft matrices. TPM values were used for clustering and visualization. **(B)** Volcano plots displaying DEGs between the two conditions based on gene expression (left panel) and fold change (right panel). CCN1 is highlighted in red. **(C)** GO enrichment analysis of genes upregulated in the 3D stiff model, categorized into BP, MF, and CC terms. **(D)** GO enrichment analysis of genes upregulated in the 3D soft model, categorized into BP, MF, and CC. Circle size represents gene count, and color indicates adjusted p-value.

To elucidate the molecular mechanisms underlying the observed phenotypic differences in stiffness-modulated cells, A549 cells cultured in 3D soft and 3D stiff models were subjected to transcriptome sequencing. The analysis revealed significant variations in gene expression profiles between the two conditions, highlighting stiffness-dependent molecular adaptations in tumor cells (Fig. 4A).

Differential gene expression analysis between stiff and soft 3D models revealed distinct transcriptional profiles. In the stiff model, significantly upregulated genes included *C19orf73*, *FOSB*, and *FITM1*. Studies have demonstrated that *FOSB* expression is significantly upregulated in chemo-resistant ovarian cancer tissue samples, suggesting that *FOSB* may serve as a potential therapeutic target for overcoming chemoresistance in ovarian cancer [45]. This finding aligns with our experimental results, which indicate that tumor cells cultured in the stiff model exhibit chemoresistance. *FITM1* has been reported to participate in lipid droplet formation, and tumors with high malignancy and metastatic potential often exhibit increased lipid synthesis and storage [46,47].

In contrast, tumor cells cultured in the soft model exhibited a broader upregulation of genes associated with proliferation and invasion, including *MKKS*, *PEDS1-UBE2V1*, *CGB8*, *LRP11*, *GNG8*, *H2AC4*, *SNX22*, *CCN1*, *AURKB*, *TK1*, *KRT6A*, *CCN2*, *ZWINT*, *LCE3D*, *HASPIN*, *CDCA3*, *IPO4*, and *SULT1A3*. Among these, CCN1 demonstrated both high expression levels and a strong fold change in the soft model relative to the stiff model, making it one of the most significantly upregulated genes identified by RNA-seq (Fig. 4B). As a member of the CCN family of matricellular proteins, CCN1 is known to regulate cell–ECM interactions and promote tumor cell migration, intratumoral angiogenesis, and ECM remodeling through collagen production and deposition [[48], [49], [50]]. The upregulation of CCN1 is consistent with the enhanced proliferation and invasive potential observed in the soft matrix model, suggesting its potential role in mediating the biomechanical regulation of tumor progression in the soft model.

Additional upregulated genes further support the proliferative phenotype in the soft model. MKKS is involved in cellular signaling regulation and may influence mitogenic signaling cascades [51]. CGB8, a member of the chorionic gonadotropin beta subunit family, promotes tumorigenesis and progression in breast cancer [52]. LRP11 has been shown to drive tumor cell proliferation and metastasis in triple-negative breast cancer via the miR-149-3p/NRP2 axis [53], and its upregulation here correlates with the observed migratory phenotype. Several proliferation-related genes were also significantly upregulated. AURKB facilitates chromosome segregation and cytokinesis, and its overexpression correlates with poor prognosis in breast, bladder, and thyroid cancers [[54], [55], [56]]. In pan-cancer analyses, *TK1* has been shown to mediate cell proliferation and promote tumor progression [57]. *KRT6* regulates the pentose phosphate pathway in NSCLC, thereby promoting tumor growth and invasion [58]. *ZWINT*, *HASPIN*, and *CDCA3* are all expressed in tumor cells to promote proliferation [[59], [60], [61]]. These transcriptional changes directly parallel the increased cell proliferation observed in the soft model. Collectively, these findings support the conclusion that ECM stiffness modulates tumor cell phenotype by regulating distinct transcriptomes.

Subsequent GO analyses of the differentially expressed genes revealed that in the stiff-cultured A549 cells, multiple metabolic pathways, including steroid metabolism, cholesterol metabolism, secondary alcohol metabolism, and regulation of oxidoreductase activity, showed significantly increased activity (Fig. 4C and D). In contrast, in soft model cultured A549 cells, activities associated with cell division, ATP hydrolysis, and DNA helicase were markedly enhanced.

The RNA sequencing results are consistent with the phenotypic differences observed between the soft and stiff models. Cells in the soft model exhibited increased proliferation, supported by upregulation of genes involved in cell division and energy production. Conversely, the stiff model promoted enhanced metabolic activity and extracellular signaling, indicative of a more secretory and metabolically active tumor state.

### 2.5 CCN1 regulates the proliferation and invasion of LC cells in soft models

**Fig. 5:**
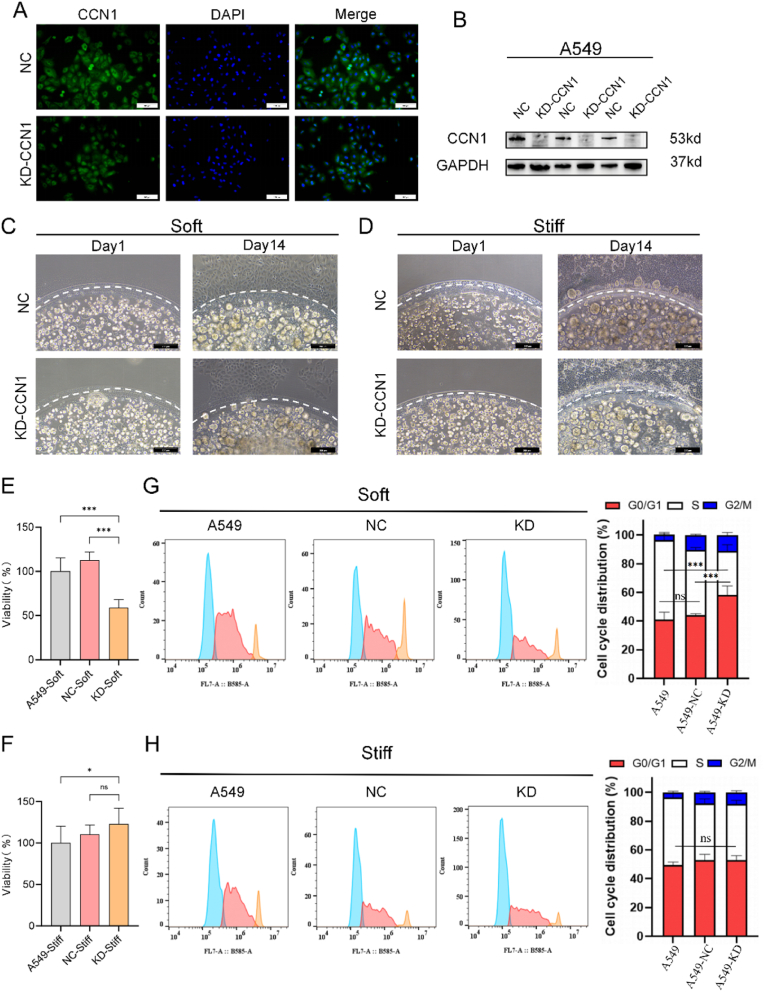
**CCN1 regulates the proliferation and invasion of LC cells in soft models. (A)** Immunofluorescence images of A549 cells with CCN1 knockdown via lentiviral transfection. Scale bar: 100 μm. **(B)** Western blot analysis confirming CCN1 knockdown efficiency in A549 cells. **(C)** Invasion of A549 cells in the negative control (NC) and CCN1 knockdown (KD-CCN1) groups under soft model culture conditions. Scale bar: 200 μm. **(D)** Invasion of A549 cells in the NC and KD-CCN1 groups under stiff model culture conditions. Scale bar: 200 μm. **(E)** Proliferation of A549 cells in the non-transfected, NC, and KD-CCN1 groups under soft model culture conditions. **(F)** Proliferation of A549 cells in the non-transfected, NC, and KD-CCN1 groups under stiff model culture conditions. **(G)** Cell cycle analysis of A549 cells in the non-transfected, NC, and KD-CCN1 groups under soft model culture conditions. **(H)** Cell cycle analysis of A549 cells in the non-transfected, NC, and KD-CCN1 groups under stiff model culture conditions. Data are presented as mean ± SD. Statistical significance: ∗p < 0.05, ∗∗∗p < 0.001; ns, not significant.

Given the enhanced proliferative capacity of LC cells cultured in soft models, we investigated the role of upregulated genes in modulating tumor cell behavior. Among the top upregulated genes in the soft model, CCN1 was identified as significantly increased. CCN1 is a matricellular protein involved in extracellular matrix remodeling and regulation of cell proliferation and differentiation. To determine the impact of CCN1 on tumor cell proliferation and invasion, we knocked down CCN1 expression (KD-CCN1) in A549 cells and compared them to negative control (NC) cells (Fig. 5A and B, Supplemenary Fig. 4), conducting experiments under both soft and stiff ECM conditions. Specifically, the untransfected group refers to A549 cells without any transfection treatment, while the NC group refers to cells transfected with a non-targeting control siRNA.

A significant reduction in the invasive capacity of A549 cells was observed in the soft model after CCN1 knockdown (Fig. 5C). This suggests that CCN1 plays a pivotal role in promoting tumor invasion in soft ECM environments. Conversely, in the stiff model, KD-CCN1 had no significant effect on invasiveness (Fig. 5D). Since CCN1 expression was already lower in the stiff model, this may indicate that CCN1's regulatory function on invasion is ECM-stiffness dependent, with a pronounced effect in softer matrices.

Consistent with previous results, tumor cells cultured in the soft model exhibited higher proliferative capacity compared to those in the stiff model. Knockdown of CCN1 expression significantly reduced proliferation in the soft model, while no notable changes were observed in the stiff model (Fig. 5E and F). Cell cycle analysis showed that CCN1 knockdown in A549 cells cultured in the soft matrix increased the G0/G1 phase population. The proportion rose from 41.1 % to 44.1 % in the untransfected and negative control (NC) groups to 58.1 % in the KD-CCN1 group. This was accompanied by a decrease in the S and G2-M phase populations (Fig. 5G). The changes were consistent across replicates, and the increase in G0/G1 was statistically significant. These results suggest that CCN1 promotes cell cycle progression under soft matrix conditions, likely by supporting the G1-to-S/G2-M transition. In contrast, CCN1 knockdown in the stiff matrix did not significantly affect cell cycle distribution (Fig. 5H). These findings suggest that CCN1-mediated regulation of cell proliferation is influenced by the mechanical properties of the tumor microenvironment.

### 2.6 Establishment and validation of patient-derived tissue (PDT) models

**Fig. 6:**
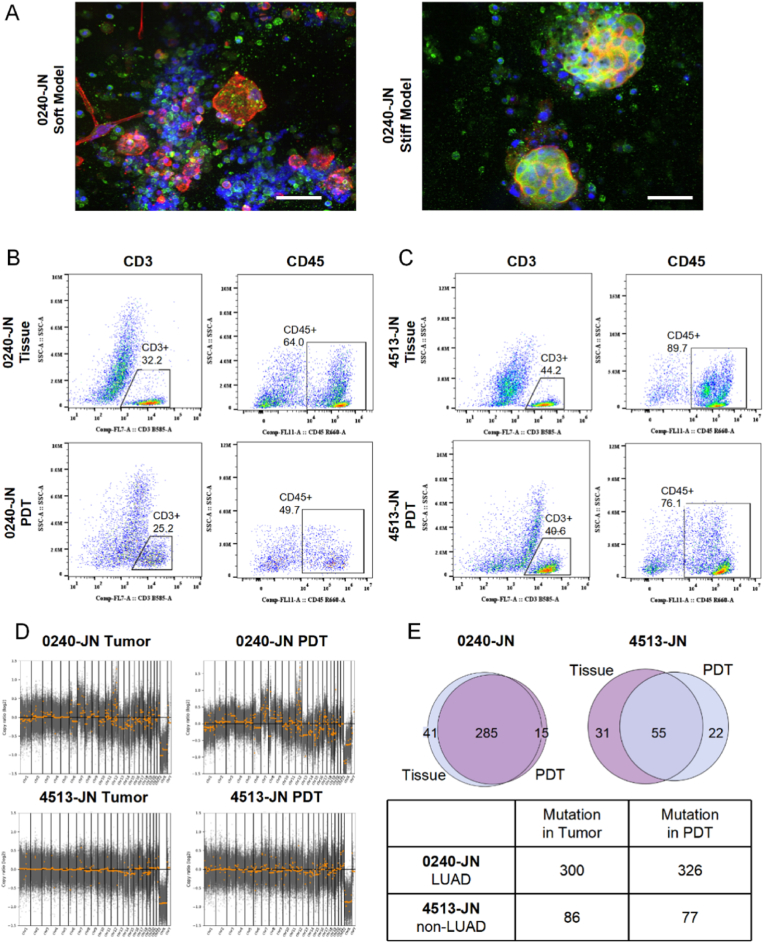
**Establishment and validation of PDT models.** **(A)** Confocal imaging of bioprinted PDTs of LUAD patient 0240-JN, with phalloidin staining (red) for the cytoskeleton and CD45 staining (green) for immune cells. Scale bar: 50 μm. **(B**–**C)** Flow cytometry analysis illustrating immune cell populations in primary tissues and bioprinted PDTs: (B) Sample 0240-JN, (C) Sample 4513-JN. **(D)** Copy number variation (CNV) analysis of primary tissue and PDTs based on whole exome sequencing results. **(E)** Venn diagram and table showing the concordance of single nucleotide variants (SNVs) between PDTs and primary tissues of representative tissues.

To establish PDT models, primary LC cells were isolated from surgically resected tumor tissues by mechanical and enzymatic digestion. The isolated cells were encapsulated in bioink and printed into disc-shaped 3D models with either soft or stiff properties. The printed models were cultured in a serum-free complete LC cell growth medium for two weeks, after which the cells were analyzed using confocal microscopy. In both primary samples, organoid-like cell spheres formed within the 3D hydrogel, and CD45^+^ immune cells were retained (Fig. 6A). Notably, some cells in the soft models exhibited a fibroblastic morphology, while those in the stiff models formed larger, more rounded spheres. We also performed flow cytometry to assess the immune cell populations, particularly CD45^+^ total immune cells and CD3^+^ T cells, which are critical components of the tumor microenvironment relevant to immunotherapy. In tumor samples from patient 0240-JN, CD45^+^ and CD3^+^ cells constituted 64.0 % and 32.2 % of the tumor tissue, respectively. The corresponding PDT model cultured ex vivo retained 49.7 % CD45^+^ cells and 25.2 % CD3^+^ cells (Fig. 6B). Similarly, in tumor samples from patient 4513-JN, CD45^+^ and CD3^+^ cells accounted for 89.7 % and 44.2 % of the tumor tissue, respectively, with the corresponding PDT model retaining 76.1 % CD45^+^ cells and 40.6 % CD3^+^ cells ex vivo (Fig. 6C). These findings suggest that the PDT models effectively preserve the tumor immune microenvironment and support tumor cell growth, capturing key characteristics of the in vivo tumor milieu.

To evaluate the fidelity of PDT models in recapitulating in vivo tumor characteristics, we compared copy number variations (CNVs) and single-nucleotide variations (SNVs) between primary cells from patient tumor samples and their corresponding PDT models. PDT models reliably reproduced CNV profiles characteristic of the original tumor tissues across different patient subtypes (Fig. 6D). For SNVs, the PDT model of patient 0240-JN, who exhibited a high mutation burden, achieved 95 % concordance in SNV profiles with the tumor tissue (Fig. 6E). For patient 4513-JN, with diagnosis of giant cell sarcoma, the PDT model demonstrated reduced similarity in SNV profiles compared to the original tissue, suggesting that PDTs may be particularly suited for modeling LUAD.

The drug responsiveness of PDTs was also evaluated. PDTs from four patients were treated with cisplatin, a platinum-based chemotherapy agent. Three PDTs demonstrated significantly greater drug sensitivity in soft matrices compared to stiff matrices, consistent with trends observed in cell-line models (Supplementary Fig. 6B). This may be attributed to higher proliferation rates in softer matrices, rendering cells more susceptible to DNA-replicating agents. However, the PDT derived from 6512-JN patient sample exhibited stiffness-independent response, emphasizing the potential impact of inter-patient heterogeneity in drug sensitivity. These findings suggest that ECM stiffness can modulate chemotherapeutic response, likely due to increased proliferation in softer matrices, while also emphasizing the potential impact of inter-patient heterogeneity in drug sensitivity.

Additionally, we tested the response of PDT immune microenvironments to anti-PD-1 antibody (αPD-1). In 0240-JN PDTs, αPD-1 treatment, at both normal and 10-fold concentrations, increased the percentage of CD3^+^ T cells and apoptotic tumor cells (Supplementary Figure 7A). Conversely, in 4513-JN PDTs, neither T cell populations nor tumor cell apoptosis increased under similar conditions (Supplementary Fig. 7B). These results suggest that PDTs can distinguish patient-specific differences in therapeutic responses.

These findings demonstrate that 3D-printed PDT models effectively replicate key features of the tumor microenvironment, including immune cell composition, genomic alterations, and drug responses. The observed stiffness-dependent variations in chemotherapy efficacy and the ability to discriminate immune responses between patients further highlight the importance of ECM properties and underscore the potential of PDTs as tools for drug sensitivity testing.

### 2.7 PDT models with immune microenvironments reflect clinical treatment outcomes

**Fig. 7:**
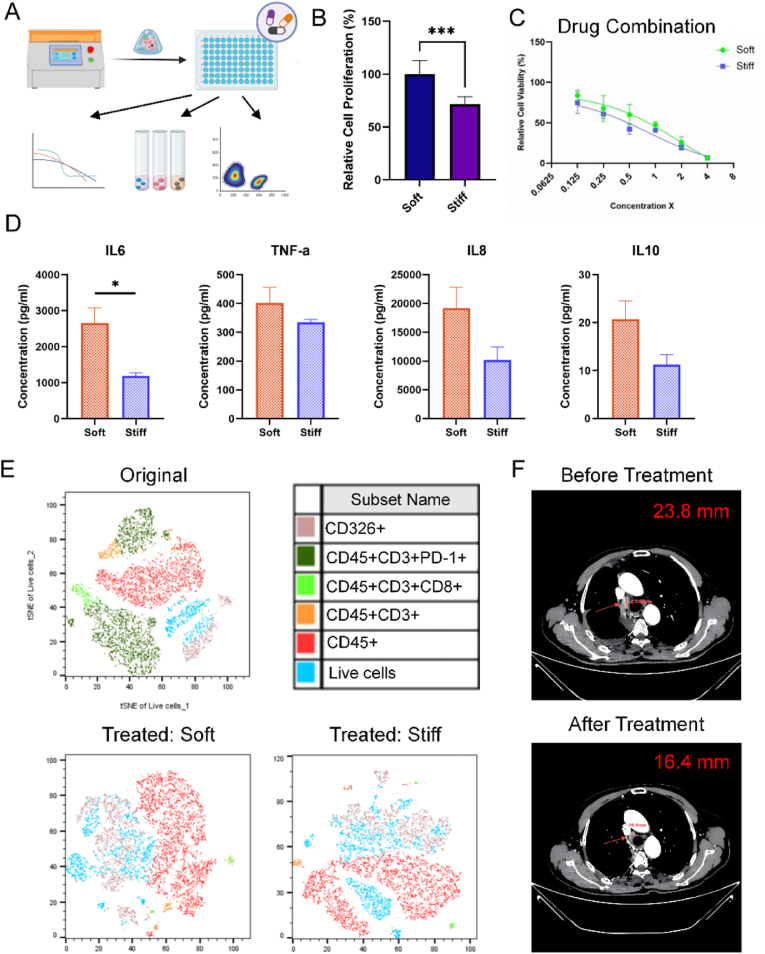
**PDT models with immune microenvironments reflect clinical treatment outcomes.** **(A)** Schematic illustration of drug sensitivity testing of combination therapy in bioprinted PDT models. **(B)** Cell proliferation in soft and stiff PDT models after 5 days of culture. **(C)** PDT viability after treatment with a combination therapy of cisplatin, pemetrexed, and sintilimab under soft and stiff conditions. **(D)** Key cytokines, including IL6, IL8, TNF-a, and IL10 measurements in supernatants collected from soft and stiff PDT models without drug treatment. **(E)** tSNE analysis of flow cytometry data showing cell population proportions in primary tissue treated soft PDT models, and treated stiff PDT models after combination therapy. **(F)** CT images showing clinical outcomes of the patient before and after treatment with combination therapy. The arrow indicates the lymph node, and the bar represents lymph node size. Data are presented as mean ± SD. Statistical significance: ∗p < 0.05, ∗∗∗p < 0.001.

To better reflect clinical treatment scenarios, we subsequently investigated the capacity of PDT models to evaluate a combination regimen of immunotherapy and chemotherapy. Using cells isolated from a representative LUAD sample (0240-JN), we constructed and cultured soft and stiff PDT models and observed that cells proliferated more extensively in the soft matrix, consistent with the trends observed in established cell line models (Fig. 7A and B). The patient's clinical treatment regimen, comprising carboplatin, pemetrexed, and an αPD-1 drug sintilimab, was applied to the PDT models in vitro. Drug concentrations were selected based on the maximum plasma concentration (C_max_) of each drug in the regimen, with 1X C_max_ serving as the baseline concentration. Drug effects on cell viability were assessed across a concentration range of 1/8X to 4X. The combination therapy demonstrated a dose-dependent response in both the soft and stiff PDTs. The IC_50_ values for the combination therapy were 0.69X in the soft model and 0.44X in the stiff model (Fig. 7C). At 1X concentration, PDT viability was below 50 % under both stiffness conditions. Microscopic analysis revealed organoid presence in the non-drug and αPD-1-treatment only groups; however, no organoid growth was observed in the combination therapy group (Supplementary Figure 8A).

Supernatants from PDT cultures, including untreated and treated groups, were collected for cytokine profiling (Fig. 7D). In untreated groups, cytokine levels indicated that matrix stiffness influences the immune microenvironment. IL-6 levels were significantly higher in the soft model compared to the stiff model, while TNF-α, IL-8, IL-10, IFN-γ, and IL-13 levels were also elevated in the soft model, though these differences were not statistically significant (Fig. 7D, Supplementary Fig. 8B). Following combination therapy, IL-13 levels were significantly reduced in both models, while IL-8 levels were elevated; the levels of other cytokines did not differ significantly between treated and untreated groups (Supplementary Fig. 8C–D). To further evaluate changes in the immune microenvironment, flow cytometry was performed on PDT models treated with 1X concentrations of the combination therapy. PD-1-positive cells, abundant in the original tissue, were reduced in the immunotherapy group compared to controls in both the soft and stiff models (Fig. 7E–Table 3). These findings indicate that immunotherapy effectively targeted PD-1 proteins on T cells, regardless of ECM stiffness, and both models displayed a similar immune response to the combination therapy.

**Table 3:** Cell population percentages in flow cytometry analysis.

Condition	%CD45+	%CD3+/CD45^+^	%CD8+/(CD45 + CD3^+^)	%PD-1+/(CD45 + CD3^+^)
Soft (Blank)	49.5	10.1	34.7	24.0
Stiff (Blank)	47.3	9.3	35.6	43.1
Soft (aPD-1)	52.6	10.3	33.4	0
Stiff (aPD-1)	49.5	6.1	39.1	0
Soft (Combination)	53.6	6.0	34.4	0
Stiff (Combination)	50.2	5.1	38.6	0

Collectively, the results demonstrated that PDTs derived from patient 0240-JN responded to the combination therapy. As the patient had undergone curative resection and had no measurable primary tumor postoperatively, disease recurrence was monitored via an enlarged mediastinal lymph node, which served as the only target lesion for response evaluation. Following the initiation of combination therapy, imaging performed in April 2024 revealed a 30 % reduction in lymph node size. This change met the threshold for partial response according to RECIST 1.1 criteria and was consistent with the in vitro treatment responses observed in the PDT models (Fig. 7F). These findings support the predictive potential of PDT models in therapeutic assessment. At the same time, the stiffness-independent drug response observed in this sample further underscores the influence of patient-specific factors and therapeutic mechanisms that may override stiffness-dependent effects. These results highlight the importance of considering both microenvironmental and intrinsic cellular factors when evaluating therapeutic efficacy in vitro.

## 3 Discussion

In this study, we utilized 3D bioprinting and biomaterials to develop NSCLC models that accurately mimic the mechanical feature of tumor ECM, providing a robust platform for investigating stiffness-driven tumor behaviors in NSCLC. These biomimetic models address the challenges posed by ECM heterogeneity and complexity, highlighting their potential to advance precision oncology by incorporating patient cells. To isolate the effect of mechanical stiffness on tumor cell behavior, we maintained constant concentrations of GelMA and HAMA across soft and stiff models while adjusting the LAP concentrations. Our findings emphasize the critical influence of ECM stiffness on tumor heterogeneity and progression. We identified significant differences in stiffness between tumor and non-tumor tissues, where softer normal tissues might create a permissive environment for invasion and expansion. LUAD tissues in general exhibited greater variability than other NSCLC subtypes. These insights informed the design of our 3D bioprinted models, which revealed that softer ECM conditions enhance tumor invasion and proliferation, while stiffer conditions promote resistance to chemotherapeutic agents like alkylating drugs. Mechanistically, ECM stiffness was found to regulate CCN1 expression, with functional studies confirming that CCN1-mediated invasion and proliferation are context-dependent.

The 3D bioprinted models demonstrated ECM stiffness-dependent variability in chemotherapeutic responses in PDTs, with notable differences in drug sensitivity observed both across patients and between different ECM conditions within the same patient (Supplementary Fig. 4B). In contrast, for combination therapies involving chemotherapy and immunotherapy, the PDT models showed no significant differences in drug response between soft and stiff matrix conditions, despite aligning with the clinical response observed for the patient (Fig. 7). This discrepancy may reflect the limited sample size, and future studies with an expanded patient cohort are planned to further assess the impact of ECM stiffness on combination therapy outcomes. These findings highlight the importance of patient-specific approaches to capture inter- and intra-patient variability in drug sensitivity, particularly under differing ECM conditions, for optimizing cancer treatment efficacy.

In summary, our work highlights the potential of bioprinted cell line-based tumor-peritumor models and PDT models as versatile tools for investigating ECM-driven tumor biology and guiding personalized therapy. By providing clinically relevant insights into tumor behaviors and drug responses, these models underscore the transformative potential of bioinspired approaches in advancing precision oncology and improving patient outcomes. Future studies that modify biomaterial compositions and concentrations alongside crosslinking parameters, expand patient sample size and subtype diversity, or integrate structural elements such as vasculature into bioprinted models could provide a more comprehensive and physiologically relevant representation of the tumor microenvironment. These advancements would enhance the translational potential of the platform and broaden its applicability across different NSCLC subtypes and disease stages. Moreover, the current study primarily focused on short-term drug responses; future work should incorporate long-term culture and drug exposure to investigate resistance mechanisms and improve the model's predictive value in guiding clinical treatment strategies.

## 4 Materials and methods

### 4.1 Ethical approval

Tumor tissues and adjacent normal tissues were collected from LC patients in the Department of Thoracic Surgery at Jiangnan University Affiliated Hospital. Informed consent was obtained from all participants, and the study was approved by the Institutional Review Board of Jiangnan University Affiliated Hospital, adhering to all applicable ethical guidelines (Approval No. LS202139).

### 4.2 Assessment of clinical sample stiffness

Fresh primary LC tissues and adjacent peritumoral tissues were sectioned into 1-mm-thick slices and placed in six-well culture plates submerged in phosphate-buffered saline (PBS). The mechanical properties of the tissues were assessed using a Piuma Nanoindenter (Optics11, Netherlands) with a 50-μm probe, following the manufacturer's protocol for submersion-based measurements. Nine distinct sites on each tissue sample were evaluated, and the mean stiffness value was calculated. A total of 17 pairs of tumor and peritumoral samples were analyzed.

### 4.3 Raman imaging of clinical samples

Raman spectroscopy was performed using a Raman confocal microspectrometer (SuperVision Medicine Bio-SV), equipped with a 532 nm solid-state excitation laser, a 50 × air objective lens, a 63 × water-immersion objective lens (Zeiss W Plan Apochromat, NA = 1), a multi-mode fiber-optic cable (10 μm diameter), and a thermoelectrically cooled charge-coupled device (CCD) detector. For all spectral acquisitions, laser power was consistently maintained at 50 mW, and spectra were collected with an integration time of 0.5 s, achieving an xy-spatial resolution of approximately 1.5 μm. The Raman spectra were recorded across a wavenumber range of 0–3600 cm^−1^. Sample spectra from patient tissues and ECM components were acquired within the spectral window of 400–3100 cm^−1^. Reference spectra for each of these components were obtained separately at a concentration of 1 mg/mL, compiled to establish a comprehensive reference spectral library for subsequent spectral regression analyses.

### 4.4 Immunohistochemical staining of clinical samples

Primary tissue samples were processed for immunohistochemical and Alcian blue staining to assess the extracellular matrix composition. Approximately 50 mg tissue pieces were fixed in 4 % paraformaldehyde solution at 4 °C overnight, washed with PBS, and dehydrated through a graded ethanol series before paraffin embedding.

For immunohistochemical staining, paraffin-embedded sections were deparaffinized with xylene and rehydrated through a graded ethanol series. Tissue sections were blocked with 3 % bovine serum albumin (BSA) for 30 min and incubated overnight at 4 °C with the primary antibody targeting Type I collagen (mouse anti-human, 1:200 dilution, Cell Signaling Technology). On the following day, sections were treated with a horseradish peroxidase (HRP)-conjugated secondary antibody (goat anti-mouse IgG, Dako K5007) and visualized using diaminobenzidine (DAB) chromogen. Cell nuclei were counterstained with Harris hematoxylin.

For Alcian blue staining, tissue sections were treated with 50 μL of Alcian blue working solution (Beyotime Biotech, China) for 1 h. Stained sections were then observed under a microscope (Olympus CKX53). The immunohistochemical staining specifically highlighted Type I collagen, while Alcian blue staining visualized hyaluronic acid.

### 4.5 Formulation and characterization of LC bioink

PBS solutions containing 10 % (w/w) GelMA (high degree of substitution, Shanghai Yuju Tech, China), 5 % (w/w) HAMA (150 kDa, Shanghai Yuju Tech, China), and 2 % (w/w) photoinitiator LAP (Shanghai Yuju Tech, China) were prepared. The final bioink composition was formulated, sterilized using a 0.22 μm filter, and aliquoted into 20 μL portions in a 24-well plate.

Using a DLP-based 3D bioprinter (Azure-12, Cyberiad Biotech, China) in single-image static mode, the bioink was printed with a thickness of 0.5 mm. Light intensity and exposure time were optimized to solidify the bioink into hydrogel constructs, which were then rinsed with 1 mL PBS and immersed in PBS. The mechanical properties of the hydrogel constructs were evaluated using a nanoindenter, employing the same methodology as for clinical sample stiffness assessment. Each hydrogel construct was tested in triplicate to ensure reproducibility. Proton Nuclear Magnetic Resonance of GelMA and HAMA were performed to confirm the successful modification upon gelatin and HA via the existence of double peaks between 5.5 and 6.5 ppm.

### 4.6 Cultivation of cell lines

The human LC cell lines A549 and H1975 were obtained from Procell. These cells were cultured in Dulbecco's Modified Eagle Medium (DMEM) supplemented with 10 % Fetal Bovine Serum and 1 % Penicillin/Streptomycin and passaged upon reaching approximately 80 % confluence.

### 4.7 Lentiviral transduction of cell lines

Lentiviral transduction of A549 cells with a fluorescent reporter was performed according to the following protocol: A549 cells were plated at a density of 1x10^5 cells per well in a 6-well plate. A lentiviral vector (HBLV-ZsGreen-Luc-Puro, HanBio Tech, China) with a multiplicity of infection (MOI) of 30 was added to the complete growth medium, supplemented with 6 μg/mL Polybrene (HanBio Tech, China) to enhance transduction efficiency. The cells were incubated for 72 h. After removal of the viral supernatant, the cells were cultured in medium containing 0.8 μg/mL Puromycin (HanBio Tech. Co. Ltd.) for an additional 48 h to select successfully transduced cells. The efficiency of transduction was assessed using fluorescence microscopy and flow cytometry.

Knockdown of CCN1 in the A549 cell line was performed according to the following protocol: A549 cells are seeded at a density of 5x10^4 cells/ml per well in a 6-well plate and cultured for 16–24 h until reaching 20–30 % confluence. A 25X concentrated infection medium is added at 40 μl per well. The volume of virus to be added is calculated based on the MOI for A549 cells (lentiviral infection MOI = 10) and the viral titer (3.5 × 10^9/mL) using the formula: virus volume = (MOI × cell count)/viral titer. After incubation for 16 h, the medium is replaced with complete growth medium and the cells are further cultured for up to 24 h. Puromycin is then added at a final concentration of 0.8 μg/mL to the complete medium and the cells are cultured for an additional 24–48 h. Subsequently, the medium is refreshed, and the infection efficiency is assessed using fluorescence microscopy and western blot analysis.

### 4.8 Transcriptome sequencing

Cells cultured in 3D bioprinted LC models after 7 days were released by digesting the hydrogels using the digestive enzyme working solution at 37 °C for 10 min. Cell suspensions were centrifuged at 400g for 5 min and the supernatants were discarded and cell pellets were resuspended with 1 mL Trizol (Thermofisher) for the extraction of RNA. Total RNA of the cells was obtained using the RNAeasy Plant Mini Kit (Qiagen) following the manufacturer's protocol, and the sequencing library was constructed with TruSeq RNA Sample Prep Kit (Illumina). The total mRNA was separated using oligo (dT) magnetic beads and interrupted into short fragments. cDNA was then synthesized from the RNA using SuperScript II Reverse Transcriptase (Invitrogen). After the library was constructed, the fragmented library was enriched by PCR amplification, and then the library was selected according to the size of the fragments. Library construction and sequencing procedures were carried out at Shanghai Honsunbio Technology.

### 4.9 Western blot (WB)

Proteins were resolved by sodium dodecyl sulfate-polyacrylamide gel electrophoresis and transferred onto a polyvinylidene fluoride membrane. To minimize non-specific interactions, the membrane was treated with QuickBlock™ Western Blocking Reagent. Subsequent incubation with a specific primary antibody against CCN1 (P4513-JN67, BD) was performed, followed by detection with a horseradish peroxidase (HRP)-conjugated goat anti-rabbit IgG (H + L) secondary antibody (A0208). Visualization of the target protein was achieved through chemiluminescence or fluorescence.

### 4.10 3D bioprinting and culture of multi-regional LC model

3D bioprinting of LC models derived from cell lines A549 and H1975 was conducted using a protocol similar to that previously described. Cells were suspended in bioink to a final concentration of 2x10^6 cells/mL, with material concentrations detailed in Table 1. Twenty microliters of the cell-laden bioink was deposited into each well of a 24-well plate, and a DLP-based 3D bioprinter (Azure-12, Cyberiad Biotechnology, China) was employed in single-image static mode to print constructs with a thickness of 0.5 mm. The composition of the bioink and light exposure parameters were optimized to print both soft and stiff scaffolds, which were then cultured in complete growth medium.

For the bioprinting of an invasion model of A549 cells post-lentiviral transduction, the first printing step remained the same as described above. Following the first printing, a second printing was conducted by applying 40 μL of acellular soft model bioink around the periphery of both soft and stiff printed constructs, utilizing printing parameters identical to those used for the soft model to create a circle-ring LC model. The inner portion of this model emulates soft and stiff tumor tissues, while the outer ring, being softer, represents the peritumoral tissue. The constructs were further cultured for an additional two weeks to monitor cellular invasion from the inner to the outer ring.

### 4.11 Staining and image acquisition

Cytoskeletal structures of 3D cultured cells within hydrogels were visualized using phalloidin-TRITC staining. The protocol involved fixing the cell cultures with 4 % paraformaldehyde for 2 h, permeabilization with 0.1 % Triton X-100 for 1 h, and subsequent washing with PBS. The cells were then incubated with phalloidin-TRITC (Beyotime Biotech, China) staining solution in the dark at room temperature for 2 h, followed by nuclear counterstaining with DAPI solution (Beyotime Biotech, China) for 5 min, and washed three times with PBS for 15 min each.

For the dual staining of CD45 immunofluorescence and cytoskeleton in 3D cells, the samples were fixed with 4 % paraformaldehyde for 2 h, permeabilized with 0.1 % Triton X-100 for 1 h, and washed with PBS. After blocking with 1 % BSA for 1 h, the samples were incubated with a primary antibody against CD45 (rabbit-anti human Polyclonal antibody, Proteintech, China) at a dilution of 1:500 overnight at 4 °C. On the following day, the primary antibody was removed, and the samples were co-incubated with phalloidin-TRITC and a secondary antibody (FITC-conjugated goat-anti rabbit IgG, Proteintech, China) for 2 h. Nuclear staining was performed with DAPI solution for 5 min, followed by three washes with PBS for 15 min each.

Bright-field and fluorescence microscopy was performed using an Olympus CKX53 microscope, while confocal imaging was conducted using an Olympus SpinSR spinning disk confocal microscope.

### 4.12 Cell cycle analysis

Cell cycle determination was conducted following the manufacturer's protocol for the assay kit (Absin Bioscience, China). Specifically, cells encapsulated within soft and stiff model hydrogels after 7 days of culture were subjected to enzymatic digestion using 200 μL of digestive enzyme working solution at 37 °C for 10 min to liberate the cells. Post-digestion, the cells were centrifuged at 1000g for 5 min, the supernatant was aspirated, and the cell pellet was fixed in prechilled 75 % ethanol at 4 °C overnight. Following centrifugation and decanting of the fixative, the cells were stained with propidium iodide staining solution and incubated at 37 °C in the dark for 30 min prior to flow cytometric analysis.

### 4.13 Isolation of cells from primary tissues

Freshly excised tumor tissues and peritumoral samples were immersed in aseptic tissue preservation solution (Miltenyi Biotech) and kept on ice, ensuring delivery to the laboratory within 24 h. Within a biosafety cabinet, the samples are rinsed three times with PBS containing 1 % Primocin (InvivoGen) and then weighed. Subsequently, 2–3 small pieces are sectioned and preserved for subsequent mechanical testing, RNA sequencing, DNA sequencing, and other analyses. The remaining tissue is minced into fragments less than 1 mm^3^ in a 7 mL centrifuge tube and treated with a digestive enzyme working solution at a ratio of 1 mL per 100 mg of tissue. The enzyme solution comprises 2 mg/mL Type I Collagenase, 1 mg/mL Hyaluronidase, and 0.1 mg/mL DNase (all from Yeasen Biotechnology, China), dissolved in DMEM/F12 medium and filter-sterilized at 0.22 μm for immediate use. Tissue fragments are incubated with the enzyme solution on a 37 °C shaker for 20–40 min, with intermittent pipetting every 15 min to facilitate digestion. Digestion is quenched with an equal volume of DMEM/F12 medium. The cell suspension is then strained through a 70 μm cell strainer, and the remaining tissue on the strainer is gently macerated with a 5 mL syringe plunger. The filtrate is flushed through the strainer with basal medium to collect the cells. The cell suspension is centrifuged at 400g for 5 min, the supernatant is aspirated, and the cell pellet is resuspended in 2 mL of red blood cell lysis buffer and incubated at room temperature for 5 min. After lysis is terminated, the cells are centrifuged again at 400g for 5 min and finally resuspended in complete growth medium for subsequent use.

### 4.14 3D bioprinting and culture of PDTs

For the 3D bioprinting of PDTs, primary cells harvested in previous sections were suspended in bioink at a final concentration of 2.5x10^7 cells/mL for the construction of printed scaffolds. Culture was conducted using a serum-free, complete growth medium specifically formulated for lung cancer PDT (CYM-LC01, Cyberiad Biotech, China).

### 4.15 Whole exome sequencing (WES)

WES analysis was conducted on both patient samples and their corresponding PDT models. DNA was extracted using the QIAamp FFPE DNA Tissue Kit (Qiagen), and its quality was evaluated through electrophoresis on a 1 % agarose gel, while concentration was quantified using the Qubit DNA Assay Kit (Life Technologies) and the Qubit 2.0 Fluorometer (Life Technologies). The DNA was fragmented to a length range of 150–230 bp, and adapters were ligated to the ends of the fragments. The DNA was then PCR-amplified and enriched using KAPA HyperExome probes (Roche). Library preparation was followed by sequencing on the Illumina NovaSeq 6000 platform (Honsunbio). Both the patient samples and PDT models were sequenced to an average depth of >400 × in the targeted exon regions.

The sequencing data was processed through various bioinformatics tools. Reads were aligned to the human reference genome (hg19) using Burrows-Wheeler Aligner v0.7.17, and duplicates were removed using Picard v1.119. Indel realignment and base quality score recalibration were performed with the Genome Analysis Toolkit v4.1.9. Somatic single nucleotide variants (SNVs) and indels were detected using Mutect2 and Varscan_Indel v2.4.2, respectively, and annotated with ANNOVAR. Copy number variations (CNVs) and gene fusions were analyzed using CNVkit v0.9.9 and Lumpy v0.2.13, respectively. Purity and ploidy were calculated manually by comparing the variant allele frequencies (VAF) of somatic SNVs with CNV log2 ratios from multiple variants within each sample. The final purity/ploidy estimates were based on Sequenza or ABSOLUTE calculations that best aligned with the manual assessments. For samples with a purity <15 %, no accurate purity estimation was possible.

To assess tumor-PDT SNV concordance, the overlap of variant calls and their respective allele fractions were compared. For each SNV detected in either the tumor or PDT, SAMTOOLS Pileup was used to calculate the VAF for both samples at the variant position, using a minimum base quality and mapping quality of 10. Variants were considered concordant if the VAF was >0 in both samples, and if they were identified by at least two variant callers in one of the samples. CNVs were compared between PDT and tumor by plotting log2 CNV values across chromosomes, with cutoffs of −0.235 and 0.2 marking deletions and amplifications, respectively, for a diploid sample with 30 % purity. Neutral regions were marked in green, while deletions and amplifications were shown in red. A narrower y-axis was used for tumor samples to enhance CNV detection. Structural variations (SVs) between PDT and tumor were visualized using LUMPY results. The SNV landscape was illustrated using the Bioconductor package GenVisR v1.8.0, and CNV heatmaps were generated using ComplexHeatmap v1.17.1.

### 4.16 Proliferation and drug sensitivity assays

Cell viabilities and proliferation were assessed using the ATP-based CellTiter-Glo 3D assay (Promega). Cells within soft and stiff model hydrogels were cultured for a specified duration, after which the supernatant was removed and replaced with 1 mL of a 0.5X dilution of the CellTiter-Glo 3D reagent. The plates were then agitated and incubated for 30 min, followed by the transfer of 100 μL of the reagent to white 96-well plates (Corning) for luminescence measurement using a Tecan Infinite E200 Pro microplate reader.

3D bioprinted cell line-derived models or PDTs were cultured for 2 days, at which point the medium was replaced with one containing a specified drug concentration. For cell line drug testing in A549 and H1975-derived 3D models, Furmonertinib and AST5902 ranging from 0.3 to 20 μM were tested for IC_50_ calculation. In the PDT models derived from patients 6512-JN, 7029-JN, 4466-JN, and 0240-JN, cisplatin was administered at a fixed concentration of 50 μM to test chemotherapy in soft and stiff models. For the PDT model of patient 0240-JN, a combination therapy comprising carboplatin, pemetrexed, and sintilimab was also tested. Drug concentrations were tested across a range of 0.125 × to 4 × the baseline (1 × ) dose. The baseline concentrations used were: carboplatin at 135 μM, pemetrexed at 306 μM, and sintilimab at 218 μg/mL. This design enabled evaluation of both individual and combination drug effects across a clinically relevant concentration spectrum.

After drug treatment for 3 days, cell viability was determined with the same CellTiter-Glo 3D (Promega) assay mentioned above and a Tecan Infinite E200 Pro microplate reader.

### 4.17 Cytokine profiline

Supernatants of the PDT cultures were collected with or without the drug. According to the Luminex Assay kit instructions, 50 μL of beads, standards or 2-fold diluted samples were added successively into the 96-well plate supplied by the kit and incubate overnight at 4 °C on a shaker at 800 rpm. After washing with the wash buffer, add 50 μL of detection antibody and incubate at room temperature on a shaker at 800 rpm for 1 h. Following another wash, add 50 μL of PE-conjugated streptavidin and incubate at room temperature on a shaker at 800 rpm for 30 min. After washing, add 100 μL of sheath fluid and incubate for 2 min. A Luminex-200 instrument was then used to read the luminescence intensity of each factor in the supernatant and convert the values according to the standard curve.

### 4.18 Clinical image acquisition and interpretation

The CT images of patient 0240-JN used in this study were obtained from the imaging system of Jiangnan University Affiliated Hospital. Patient 0240-JN underwent surgical treatment for invasive adenocarcinoma of the right upper lung in May 2023. Two months postoperatively, enlargement of the mediastinal lymph nodes was detected, with the largest node measuring 23.8 mm in diameter. Following two cycles of treatment with carboplatin, pemetrexed, and sintilimab in April 2024, a follow-up CT scan revealed a reduction in the size of the mediastinal lymph nodes, with the largest diameter decreasing to 16.4 mm.

### 4.19 Statistical analysis

Data on cell proliferation and drug sensitivity tests were obtained in quadruplicate and analyzed using GraphPad software. The IC_50_ values of cells within PDT under the influence of drugs were derived from the nonlinear regression of data obtained from the CellTiter-Glo 3D assay. Significance analysis was performed using one-way ANOVA, with P < 0.05 considered statistically significant. Data on biomarker detection by Luminex Assay was conducted in triplicates.

## CRediT authorship contribution statement

**Shiwei Xu:** Writing – review & editing, Writing – original draft, Visualization, Methodology, Investigation, Formal analysis, Data curation. **Xin Sun:** Visualization, Methodology, Formal analysis. **Yexin Gu:** Writing – original draft, Visualization, Methodology, Investigation, Formal analysis, Data curation. **Tong Liu:** Investigation. **Shiyin Liu:** Investigation. **Yuan Weng:** Investigation. **Weimin Zhang:** Investigation. **Leisheng Wang:** Investigation. **Mengzhen Zhou:** Methodology. **Guye Lu:** Methodology. **Min Tang:** Writing – review & editing, Methodology, Funding acquisition, Conceptualization. **Haifeng Wang:** Funding acquisition, Conceptualization. **Jinyou Li:** Writing – review & editing, Funding acquisition, Conceptualization.

## Declaration of generative AI and AI-assisted technologies in the writing process

During the preparation of this work the authors used ChatGPT to improve language confluency. After using this tool/service, the authors reviewed and edited the content as needed and took full responsibility for the content of the publication.

## Funding

The work was supported by Wuxi Taihu Lake Talent Plan which supports for Leading Talents in Medical and Health Profession (453210903THGD), General project of 10.13039/501100004608Natural Science Foundation of Jiangsu Province (BK20221204), and 10.13039/501100001809National Natural Science Foundation of China (32301149).

## Declaration of competing interest

The authors declare the following financial interests/personal relationships which may be considered as potential competing interests:Min Tang reports financial support was provided by 10.13039/501100001809National Natural Science Foundation of China. Jinyou Li reports financial support was provided by Wuxi Taihu Lake Talent Plan. Jinyou Li reports financial support was provided by 10.13039/501100004608Natural Science Foundation of Jiangsu Province. If there are other authors, they declare that they have no known competing financial interests or personal relationships that could have appeared to influence the work reported in this paper.

## Data Availability

Data will be made available on request.
