# Supplementary material for: ECM characterization and 3D bioprinted models of NSCLC for investigating stiffness-dependent tumor behavior and drug response
Source: Mater Today Bio. 2025 Apr 30;32:101823. doi: 10.1016/j.mtbio.2025.101823 (PMC12098162; doi:10.1016/j.mtbio.2025.101823)
Supplement: Multimedia component 1 [file mmc1.docx]

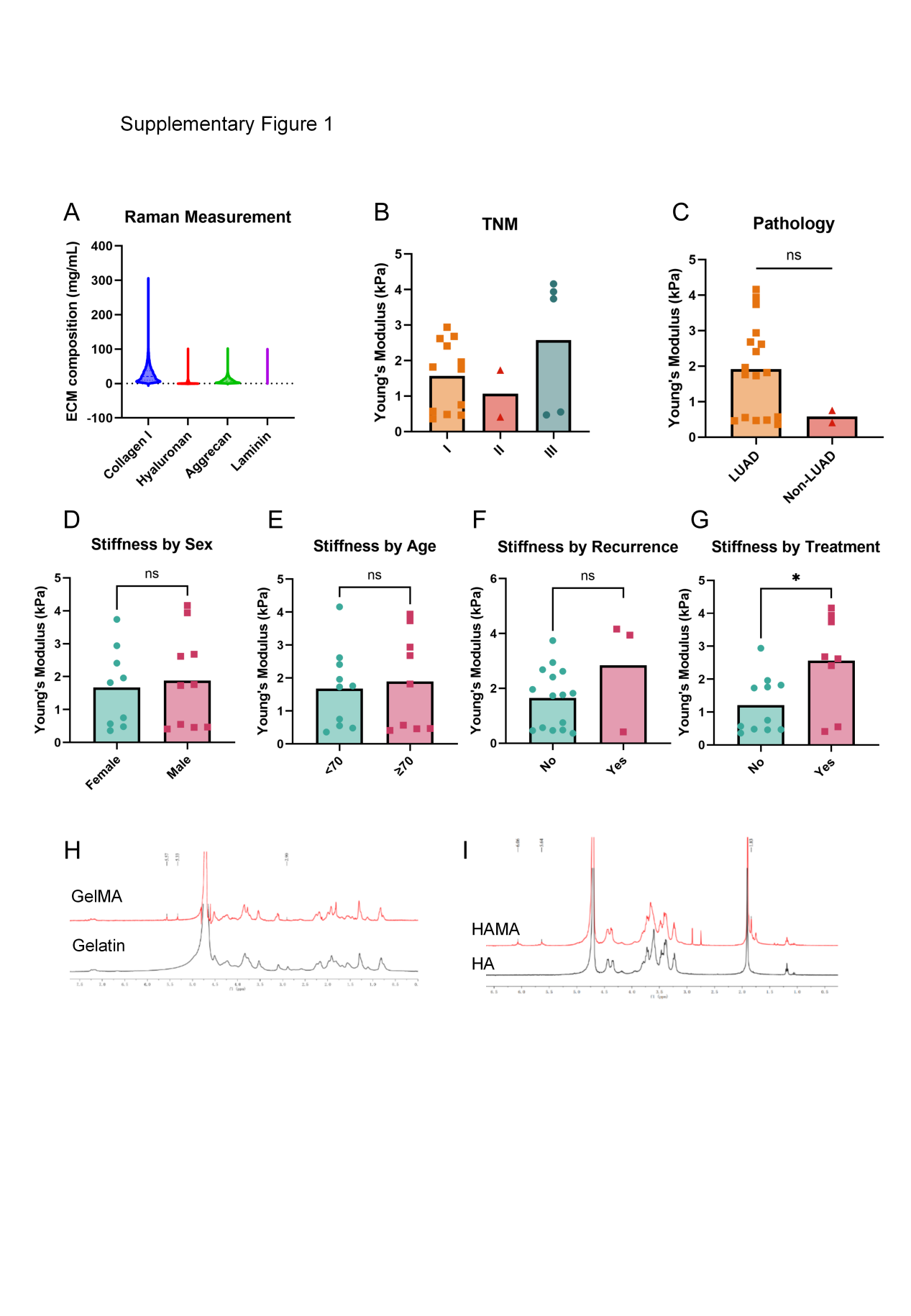


**Supplementary Figure 1.**

(A) Raman quantification of different ECM components in NSCLC tissue. (B) Stiffness distribution categorized by TNM classification of patients. (C) Stiffness distribution categorized by pathology classification of patients, with LUAD representing lung adenocarcinoma. (D) Stiffness distribution categorized by sex. (E) Stiffness distribution categorized by age. (F) Stiffness distribution categorized by recurrence. (G) Stiffness distribution categorized by treatment. (H) NMR spectra of GelMA and gelatin. (I) NMR spectra of HAMA and HA. Data are presented as mean ± SD. Statistical significance: * p<0.05, ns, not significant.


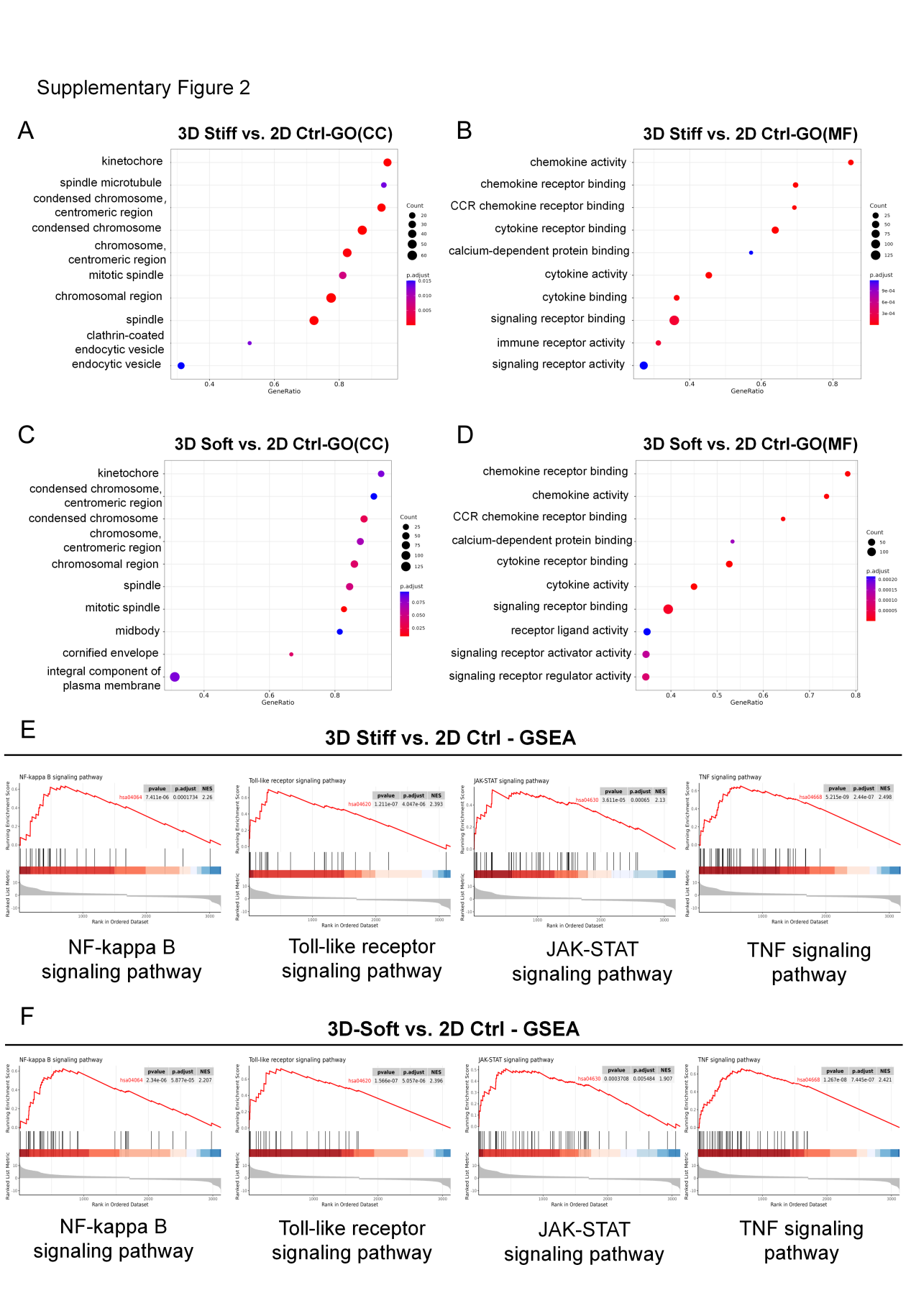


**Supplementary Figure 2.**

(A) Gene Ontology (GO) enrichment analysis of cellular component (CC) terms for genes with increased expression in A549 cells cultured in the 3D stiff model compared to the 2D culture group. (B) GO enrichment analysis of molecular function (MF) terms for genes with increased expression in A549 cells cultured in the 3D stiff model compared to the 2D culture group. (C) GO enrichment analysis of cellular component (CC) terms for genes with increased expression in A549 cells cultured in the 3D soft model compared to the 2D culture group. (D) GO enrichment analysis of molecular function (MF) terms for genes with increased expression in A549 cells cultured in the 3D soft model compared to the 2D culture group. (E) Gene set enrichment analysis (GSEA) plot of gene pathways with increased expression in A549 cells cultured in the 3D stiff model compared to the 2D culture group. (F) GSEA plot of gene pathways with increased expression in A549 cells cultured in the 3D soft model compared to the 2D culture group.


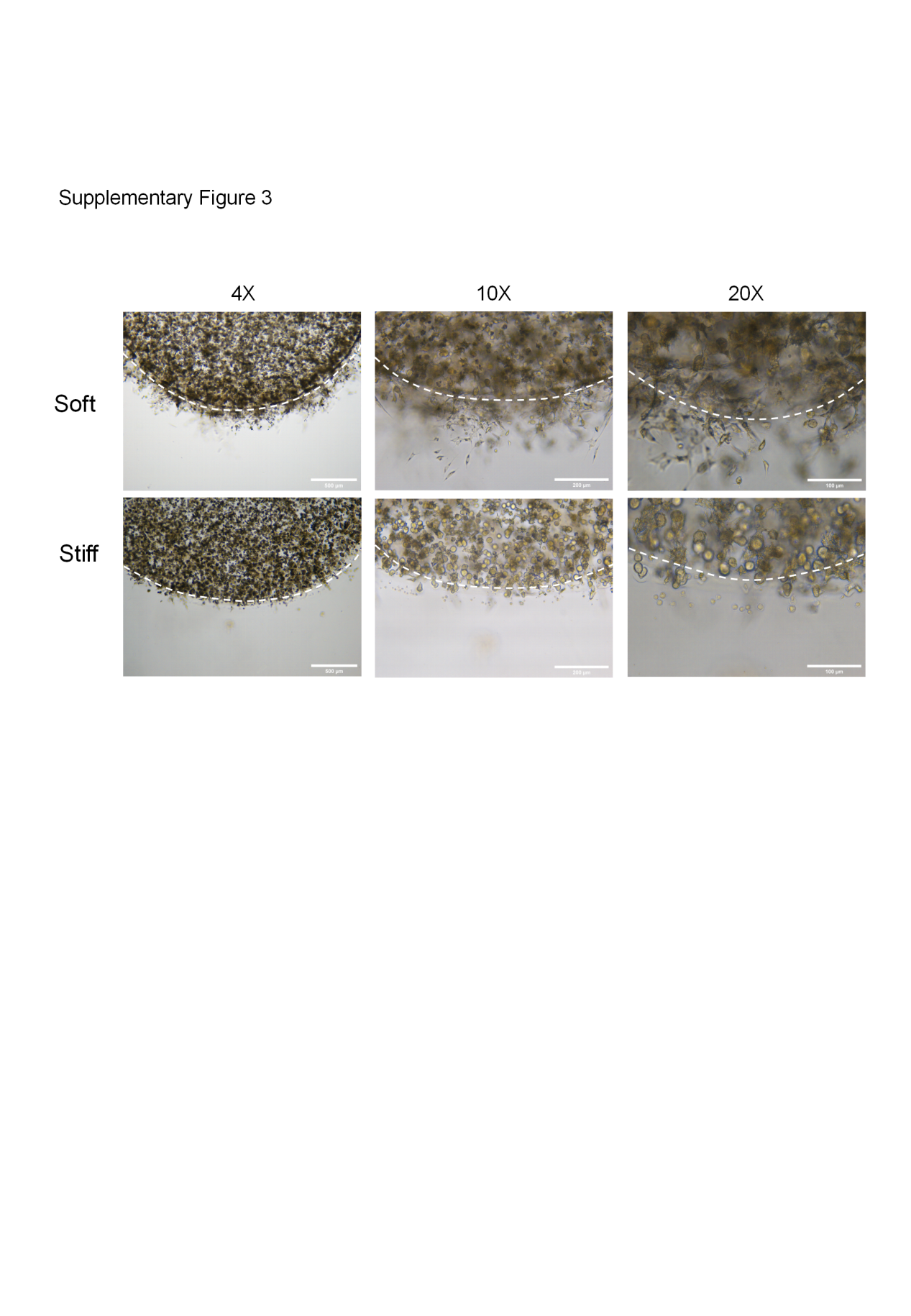


**Supplementary Figure 3.**

Invasion images of H1975 cells cultured in 3D soft and stiff tumor-peritumor models, with scale bars representing 500 μm, 200 μm, and 100 μm, respectively.


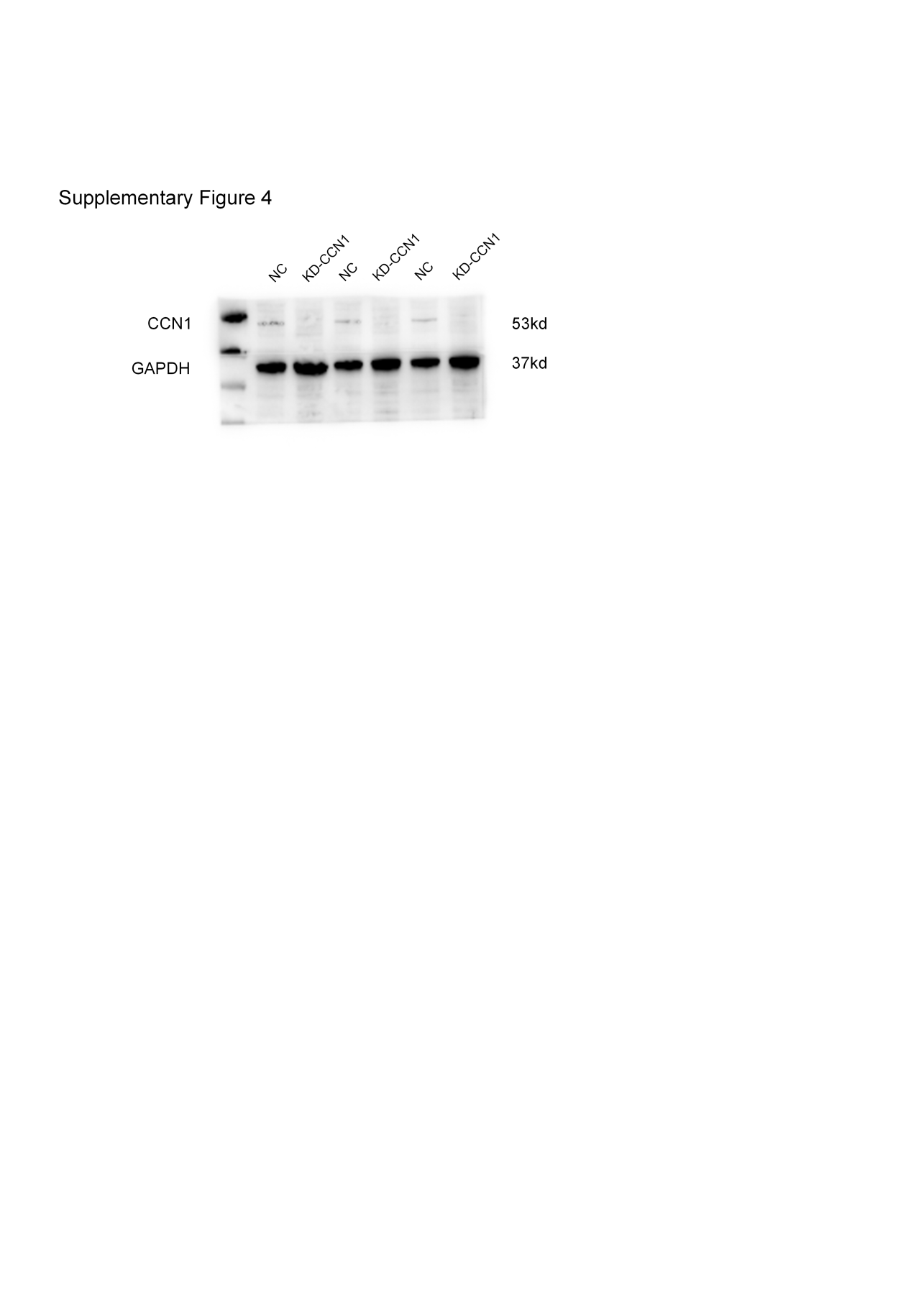


**Supplementary Figure 4.**

Full-length Western blot image showing protein levels of CCN1 in negative control (NC) and CCN1 knockdown (KD-CCN1) groups across three biological replicates. GAPDH serves as a loading control.


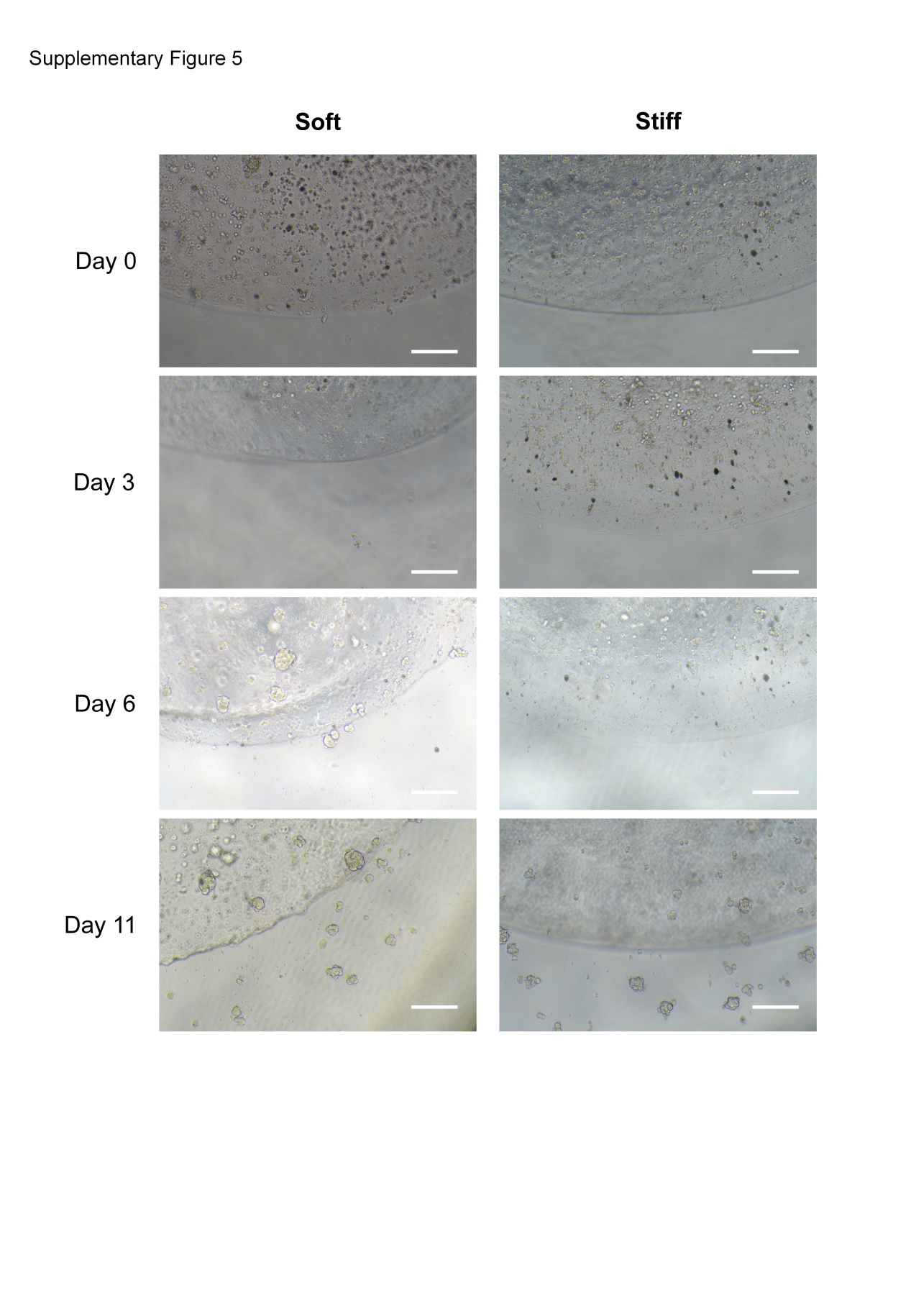


**Supplementary Figure 5.**

Representative bright-field images showing the morphological evolution of primary tumor cells cultured within soft and stiff 3D PDT models over 11 days. Images were acquired on Day 0, 3, 6, and 11 to monitor cell aggregation and organoid formation. Scale bar: 200 μm.


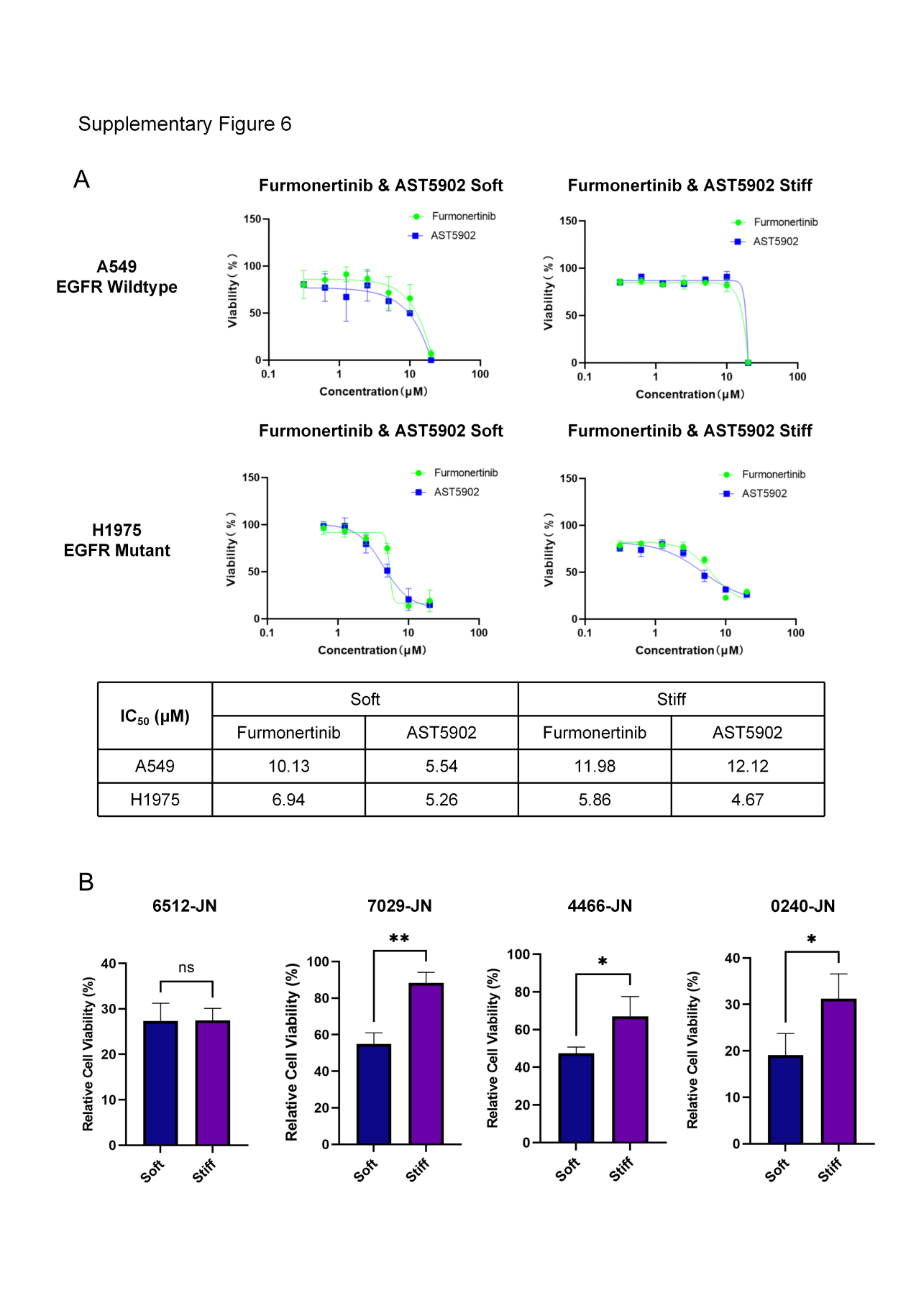


**Supplementary Figure 6.**

(A) IC50 measurement of the EGFR-targeted drug Furmonertinib and its principal metabolite AST5902 in A549 and H1975 cells. (B) Cell viability of four PDT models under treatment of chemotherapy drug cisplatin at 50 μM. Data are presented as mean ± SD. Statistical significance: * p<0.05, **p < 0.01; ns, not significant.


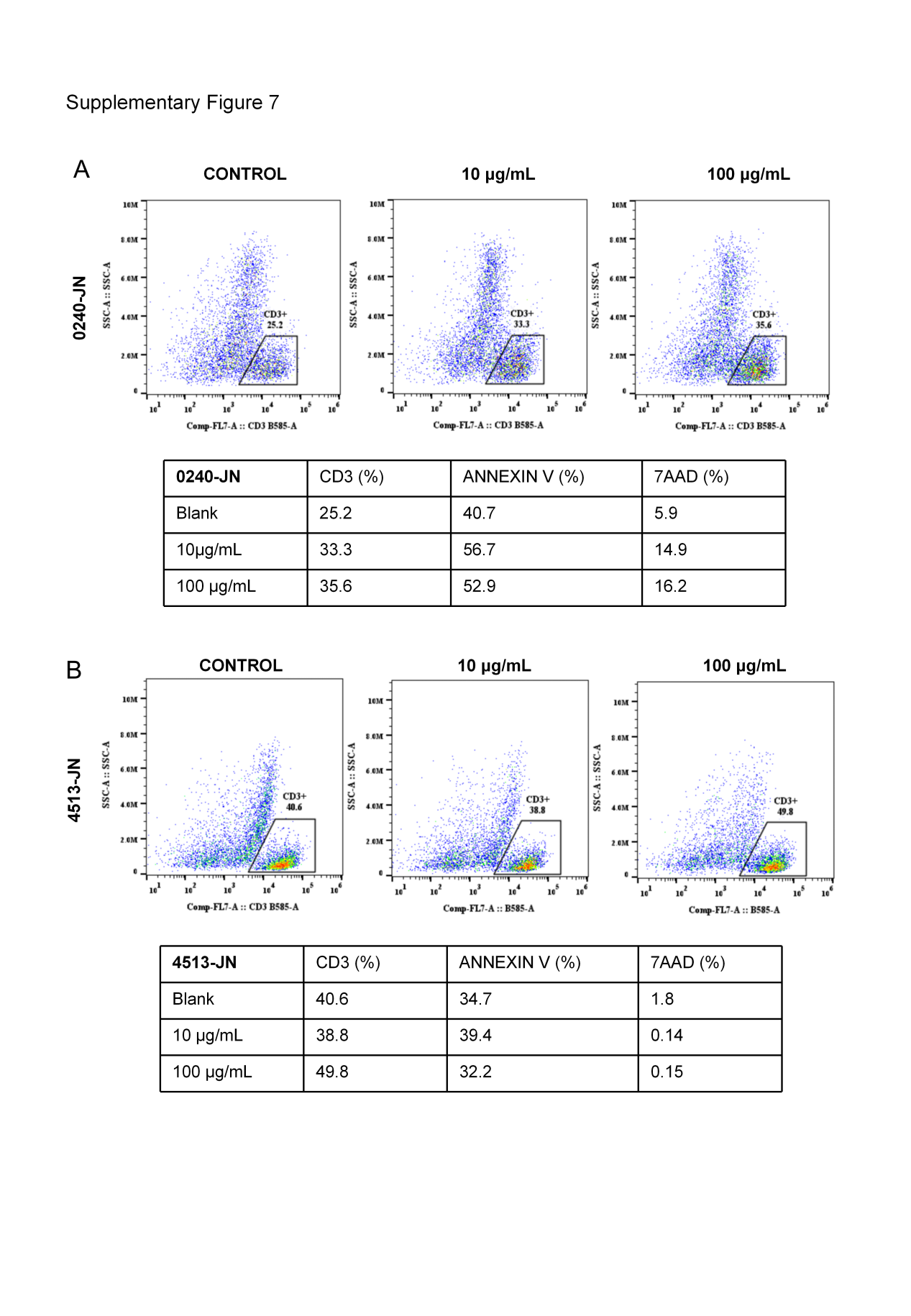


**Supplementary Figure 7.**

Flow cytometry analysis showing the percentage of CD3+ T cell populations and the percentage of apoptotic cells assessed using Annexin V and PI staining in PDTs of (A) 0240-JN and (B) 4513-JN treated with no drug, 10 μg/mL aPD-1, and 100 μg/mL aPD-1.


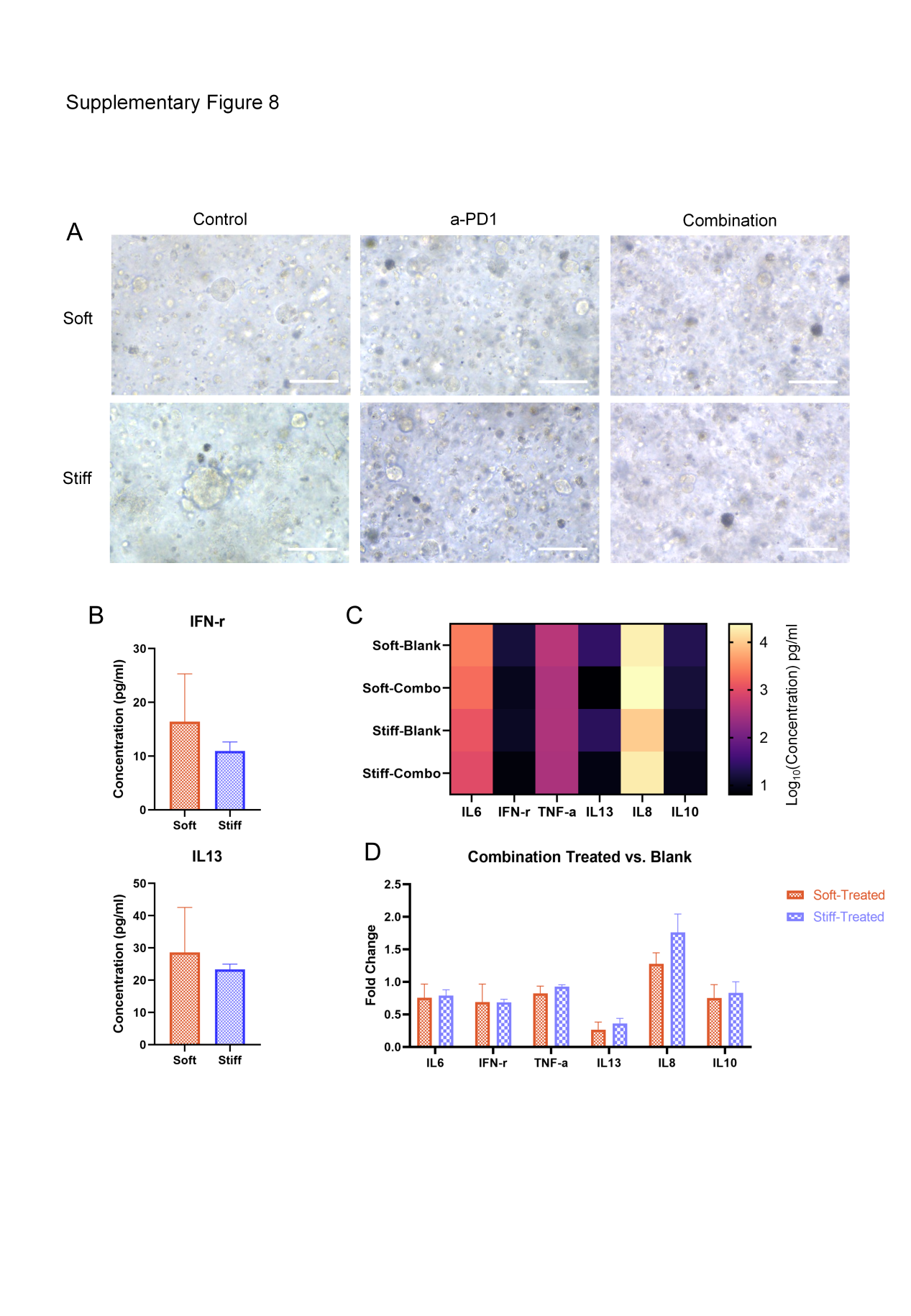


**Supplementary Figure 8.**

(A) Bright-field images of 0240-JN PDTs cultured in soft and stiff models under no-drug, aPD-1, and combination therapy treatments. Scale bar represents 100 μm. (B) Cytokine measurements of IFN-γ and IL-13 in supernatants collected from soft and stiff PDT models without drug treatment. (C) Cytokine concentrations with and without the combination therapy treatment in soft and stiff PDT models. Blank: no drug treatment. Combo: combination therapy treatment. (D) Fold changes of cytokine concentration after combination therapy treatment in soft and stiff PDT models. Data are presented as mean ± SD.
